# Circulating microRNA signatures for echinococcosis

**DOI:** 10.1186/s13071-025-07161-8

**Published:** 2025-12-01

**Authors:** Rui Li, Xuedong He, Shuangjuan Liu, Guanghui Zheng, Jing Zhang, Yongjie Kou, Xiaojiao Li, Xiaola Guo, Xiangwen Xu, Qingming Kong, Pengfei Cai, William C. Cho, Yadong Zheng, Wenhui Wang, Xueyong Zhang, Houhui Song

**Affiliations:** 1 College of Veterinary Medicine, Gansu Agricultural University, Lanzhou, 730070 China; 2 Key Laboratory of Applied Technology On Green-Eco-Healthy Animal Husbandry of Zhejiang Province, Zhejiang Provincial Engineering Laboratory for Animal Health Inspection & Internet Technology, Zhejiang International Science and Technology Cooperation Base for Veterinary Medicine and Health Management, Belt and Road International Joint, Laboratory for One Health and Food Safety, College of Animal Science and Technology & College of Veterinary Medicine of Zhejiang A&F University, 666 Wusu Street, Lin’an District, Hangzhou, 311300 China; 3 Medical Laboratory, Weinan Central Hospital, Weinan, 714000 Shaanxi People’s Republic of China; 4 National Institute of Parasitic Diseases, Chinese Center for Disease Control and Prevention (Chinese Center for Tropical Diseases Research), Key Laboratory on Parasite and Vector Biology, Ministry of Health; WHO Centre for Tropical Diseases; National Center for International Research on Tropical Diseases‚ Ministry of Science and Technology, Shanghai, 200025 China; 5 BioBank, The First Affiliated Hospital of Xi’an Jiaotong University, Xi’an, 710061 Shaanxi China; 6 State Key Laboratory for Animal Disease Control and Prevention, Key Laboratory of Veterinary Parasitology of Gansu Province, Lanzhou Veterinary Research Institute, Chinese Academy of Agricultural Sciences (CAAS), Lanzhou, 730046 Gansu China; 7 School of Laboratory Medicine and Bioengineering, Key Laboratory of Biomarkers and In Vitro Diagnosis Translation of Zhejiang Province, Zhejiang Provincial People’s Hospital (Affiliated People’s Hospital)—Hangzhou Medical College, Hangzhou, 310013 China; 8 Molecular Parasitology Laboratory, QIMR Berghofer Medical Research Institute, Brisbane, QLD Australia; 9 Department of Clinical Oncology, Queen Elizabeth Hospital, Hong Kong, SAR China; 10 Academy of Animal and Veterinary Sciences, Qinghai University, Xining, 810016 Qinghai Province China

**Keywords:** Echinococcosis, *Echinococcus multilocularis*, MiR-192-5p, MiR-122-5p, MiR-21a-5p, Diagnostic and prognostic signature

## Abstract

**Background:**

Echinococcosis, a serious zoonotic parasitic disease caused by tapeworms of the genus *Echinococcus*, is clinically characterized by a long latent period of up to 10 years. An accurate diagnosis is critical for the efficient management and treatment of patients. The aim of this study was to identify robust diagnostic signatures for echinococcosis.

**Methods:**

Using co-immunoprecipitation and RNA sequencing (RNA-seq), we comparatively profiled the Argonaute 2-binding microRNAs (abmiRNAs) in hepatocytes of *Echinococcus multilocularis*-infected mice. Using receiver operating characteristic curve (ROC) analysis, we established quantitative PCR (qPCR) assays based on circulating liver-specific abmiRNAs and their combinations, and further validated these assays using blinded serum samples from infected mice and patients.

**Results:**

Three abmiRNAs were identified as being predominantly expressed in the liver: miR-192-5p, miR-122-5p and miR-21a-5p. While these three abmiRNAs are upregulated in liver cancer and hepatitis virus infections, we found that all circulating abmiRNAs were significantly and gradually downregulated as the *Emultilocularis* infection progressed; however, they were rapidly upregulated following anthelmintic treatment. The qPCR assays targeting these circulating abmiRNAs and their combinations showed high sensitivity and specificity. Individual circulating abmiRNAs and their combinations accurately distinguished infections in both mice (*n* = 50) and humans (*n* = 117), with the combination of miR-192-5p and miR-122-5p being particularly effective in distinguishing infections. This combination was also sensitive to anthelmintic treatment.

**Conclusions:**

These results suggest that circulating miR-192-5p and miR-122-5p are serum signatures for the diagnosis and prognostic management of echinococcosis.

**Graphical Abstract:**

**Figure:**
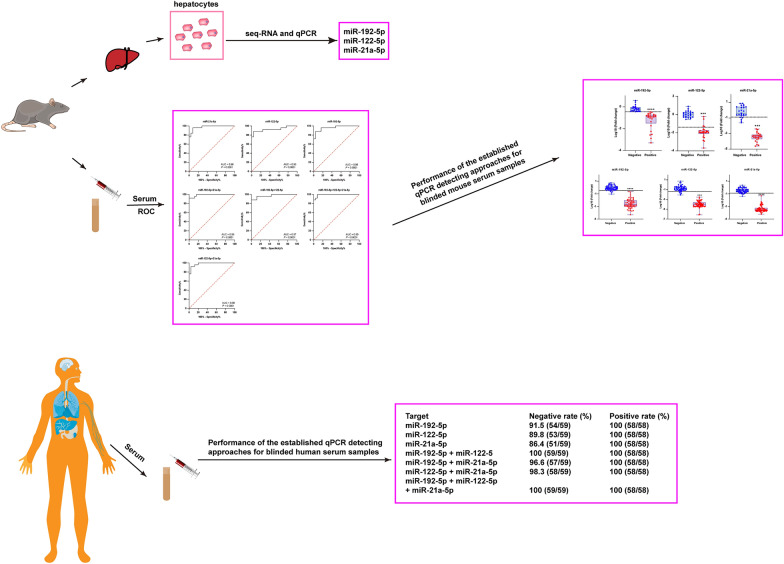

**Supplementary Information:**

The online version contains supplementary material available at 10.1186/s13071-025-07161-8.

## Background

Echinococcosis is one of the neglected tropical diseases recognized by the WHO. The two main causal agents are the tapeworms *Echinococcus granulosus* and *Echinococcus multilocularis*, which are responsible for cystic echinococcosis (CE) and alveolar echinococcosis (AE), respectively [1, 2]. CE has a global distribution, with an annual incidence rate of 1–200 cases per 100,000 in endemic areas, which is significantly higher than that of AE (0.03–1.2 cases per 100,000) [3, 4]. However, the mortality rate for CE is significantly lower than that of AE, which has a 10-year mortality rate of 94% when untreated [4]. Echinococcosis generally has a long asymptomatic period of up to 10 years, leading most patients to seek treatment only when the disease reaches an advanced stage. The treatments primarily involve surgical intervention and the administration of anthelminthic drugs, with the latter associated with considerable side effects [5, 6]. However, the frequent occurrence of multiple large lesions in the liver, which extensively invade critical intrahepatic structures such as blood vessels and bile ducts, complicates surgical procedures and reduces the effectiveness of anthelminthic therapy, often resulting in a poor prognosis [7, 8]. For patients receiving only with anthelminthic therapy, rational use is a major concern due to the considerable side effects of these drugs. Therefore, a precise diagnosis and assessment of drug efficacy are key steps in the prognosis and treatment management of echinococcosis. Clinically, the diagnosis of echinococcosis largely depends on conventional imaging technologies, which place high demands on both equipment and operators and are also limited in detecting small cysts < 2 cm in diameter [9–11]. Therefore, to efficiently control echinococcosis, reliable non-invasive diagnosis technologies are urgently needed.

MicroRNAs (miRNAs) are a type of non-coding regulatory RNA that are loaded into the Argonaute-orchestrating RNA-induced silencing complex and involved in the posttranscriptional regulation of target genes [12]. An increasing number of studies have demonstrated that the levels of specific miRNAs circulating in the blood and other body fluids of patients are closely correlated with disease status, and thus they can serve as theranostics targets [13]. In a mouse model of drug-induced liver injury, the levels of serum miR-122 and miR-192 were significantly elevated in a dose-dependent manner and were highly synchronized with changes in alanine aminotransferase activity in sera, demonstrating for the first time the feasibility of circulating miRNAs as biomarkers of liver injury [14]. Similarly, in the field of non-alcoholic fatty liver disease, the differential expression of serum miR-122, miR-192 and miR-375 was used to effectively distinguish non-alcoholic steatohepatitis from simple steatosis [15]. In hepatitis B virus and hepatitis C virus infections, miR-122 was also found to be significantly upregulated and negatively correlated with viral load and necroinflammation, suggesting its potential as a marker for these viral infections [16, 17]. During *Leishmania* infection, hepatocytes can take up the exosomes containing parasite membrane-derived zinc-dependent metalloprotease GP63, which are released from infected mononuclear phagocytes [18]. The results of this study further demonstrated that GP63 induces the proteolytic inactivation of Dicer 1, thereby blocking the processing of pre-miRNAs, including pre-miR-122, into mature miRNAs. The loss of miR-122 in the liver may increase parasitic burden in patients with visceral leishmaniasis, suggesting that miR-122 is a promising therapeutic target [18].

To define reliable non-invasive signatures for echinococcosis, we profiled Argonaute 2-binding miRNAs (abmiRNAs) in the hepatocytes of mice experimentally infected with AE and identified three abmiRNAs circulating in the blood that were highly associated with the disease status of AE. Finally, we systematically assessed the reliability of these three circulating abmiRNAs as diagnostic and prognostic biomarkers.

## Methods

### Parasite infection and sample collection

*Echinococcus multilocularis* Qinghai isolate was maintained in Mongolian gerbils (*Meriones unguiculatus*) in our laboratory, and protoscoleces were recovered as previously described [19]. Six-week-old BALB/c mice were purchased from Hangzhou Medical University (Hangzhou, PRC). The infection group was intraperitoneally injected with approximately 1000 protoscoleces per mouse, while the control group received normal saline. All animals were anesthetized using carbon dioxide at 30, 60 and 90 days post-infection, and their sera and livers were collected under sterile conditions for further analysis. The hepatocytes were isolated as previously reported [20] and were immediately used or stored at − 80 °C.

### Sera from healthy humans and patients with echinococcosis

Patients with echinococcosis were diagnosed by multiple approaches, including ultrasound, enzyme-linked immunosorbent assay (ELISA) and postoperative pathology, as previously described [21]. A total of 59 and 58 serum samples were collected from healthy individuals and patients, respectively, and the samples were immediately stored at − 80 °C until use.

### Total RNA extraction and high-throughput sequencing

Total RNA was extracted using TRIzol reagent (Invitrogen, Thermo Fisher Scientific, Waltham, MA, USA) following the manufacturer’s instructions. DNase I (New England Biolabs [NEB], Ipswich, MA, USA) was used to prevent genomic DNA contamination. Total RNA was extracted from 100 μl of mouse sera or 30 μl of human sera using the miDETECT™ miRNA External Control product (which contains* Caenorhabditis elegans* miR-39-3p as external reference gene; RiboBio Co., Ltd., Guangzhou, PRC). Following quality analysis using electrophoresis and NanoDrop spectrophotometry (Thermo Fisher Scientific), the RNA samples were immediately stored at − 80 °C until use in subsequent experiments.

To profile abmiRNAs in the hepatocytes, we processed the RNA samples as previously reported [22] and forwarded them for sequencing by BGI Genomics (Yantian, Shenzhen, PRC). AbmiRNAs were identified as previously described [23]. Briefly, clean reads were mapped to the mouse genome (http://hgdownload.cse.ucsc.edu/goldenPath/mm10/bigZips/) after the removal of contaminated sequences. Known and novel miRNAs were defined, and the differentially expressed abmiRNAs were screened under the conditions: |log2 fold change| > 1 and *Q*-value < 0.001.

### Quantitative real-time PCR

A 1-µg aliquot of total RNA was reversely transcribed into complementary DNA (cDNA) using the All-in-One™ miRNA First-Strand cDNA Synthesis Kit (Genecopoeia, Rockville, MD, USA) according to the manufacturer’s instructions. Quantitative PCR (qPCR) was performed using the miScript SYBR Green PCR Kit (Vazyme, Nanjing, PRC) on the Mx3000P system (Agilent Technologies, Santa Clara, CA, USA) under the following conditions: 95 °C for 30 s, followed by 40 cycles of 95 °C for 10 s and 60 °C for 30 s. The relative expression changes were calculated using the 2^−ΔΔCt^ method, with cel-miR-39-3p as the reference. Each sample was set in triplicate, and the specific primers are listed in Additional file 1: Table S1).

### Construction of standard curves

To assess the efficiency and sensitivity of the qPCR analyses, recombinant plasmids containing either miR-192-5p, miR-122-5p or miR-21a-5p, were constructed. Briefly, PCR reactions were conducted using the A9 LongHiFi PCR Master Mix (Aidlab Biotechnologies, Beijing, PRC), and the amplicons were cloned into the pTOPO-Blunt Cloning Vector (Aidlab Biotechnologies). The recombinant plasmids were extracted using a high-purity plasmid extraction kit (HLingene Biotechnology Ltd., Shanghai, PRC) and sent for sequencing to the gene technology company (Qingke Biology, Qingdao, Shandong, PRC). The copy number of each plasmid sample was determined as previously reported [24, 25].

Serial dilutions (1 × 10^10^ to 1 × 10^2^ copies/μl) of the recombinant plasmids were used to construct individual standard curves. qPCR was conducted using the All-in-One qPCR Mix Kit (Genecopoeia) on the Mx3000P system (Agilent Technologies) applying the following protocol: 95 °C for 5 min, followed by 39 cycles of 95 °C for 15 s and 60 °C for 1 min. The qPCR efficiency was calculated as previously reported [26].

### Western blotting and ELISA

Total proteins were extracted from the hepatocytes using RIPA Lysis Buffer (NCM Biotech, Newport, RI, USA) supplemented with protease inhibitors (1:100; Sigma, St. Louis, MO, USA), followed by measurement of the concentration using the BCA Protein Assay Kit (Beyotime Biotechnology, Shanghai, PRC). A 35-µg aliquot of proteins was separated by 8% sodium dodecyl sulfate-polyacrylamide gel electrophoresis (SDS-PAGE) (Vazyme) and loaded onto 0.45-μm PVDF membranes (MilliporeSigma, Burlington, MA, USA) by wet transfer at 150 V. The membranes were blocked in TBST containing 5% non-fat milk for 2 h at room temperature and then incubated with anti-Argonaute 2 antibodies (1: 1000; Abcam, Cambridge, UK) overnight at 4 °C. After three 5-min washes with TBST, the membranes were incubated with horseradish peroxidase (HRP)-conjugated anti-rabbit secondary antibodies (1:10,000; Sigma) for 1 h at room temperature. Signals were detected using the MiniChemi 910 Chemiluminescence Imager (Sagecreation, Beijing, PRC).

Ninety-six-well plates (Corning Inc., Corning, NY, USA) were coated with recombinant Em-18 (1 μg/ml) previously prepared in our laboratory [21], incubated overnight at 4 °C and then blocked with 300 μl of casein blocking buffer (B6429) for 1 h at 37 °C. After three washes in PBST, the plates were incubated with 100 μl of mouse sera (1:800) at 37 °C for 1 h, followed by incubation with 100 μl of HRP-conjugated goat anti-mouse immunoglobulin G (IgG; 1:8000; Jackson immuno research, USA) at 37 °C for 1 h. After washing 3 times with 300 μl of PBST, the reactions were developed by adding 100 μl of TMB One Solution (Promega, Madison, WI, USA) for 7 min in the dark. The reactions were stopped with 50 μl of 2 M H_2_SO_4_, and the optical density (OD) at 450 nm was measured using a BioTek Synergy H1 microplate reader (Agilent Technologies). The P/N ratio (OD value of each test serum [P] divided by the OD value of control serum [N]) was calculated. Samples with P/N > 2 were considered to be positive. All samples were run in triplicate.

### Statistical analysis

Statistical analysis was conducted using Prism 10 software (GraphPad Software, San Diego, CA, USA). Student’s t-test was used for comparisons between two groups, while one-way analysis of variance (ANOVA) was applied for three or more groups, followed by post hoc multiple comparisons by the Bonferroni test. Fisher’s exact test was used to evaluate differences among qPCR detection approaches. The association between infected and control sera was assessed by calculating the odds ratio (OR) with a 95% confidence interval (CI) using logistic regression. The receiver operating characteristic (ROC) curve was used to analyze the sensitivity and specificity of the qPCR approaches. Differences in diagnostic performance between individual miRNAs and their combinations were evaluated using logistic regression models in SPSS (SPSS IBM, Armonk, NY, USA), with *P* < 0.05 considered to be statistically significant.

## Results

### AbmiRNA profiles are altered in response to *E. multilocularis* infection

**Fig. 1:**
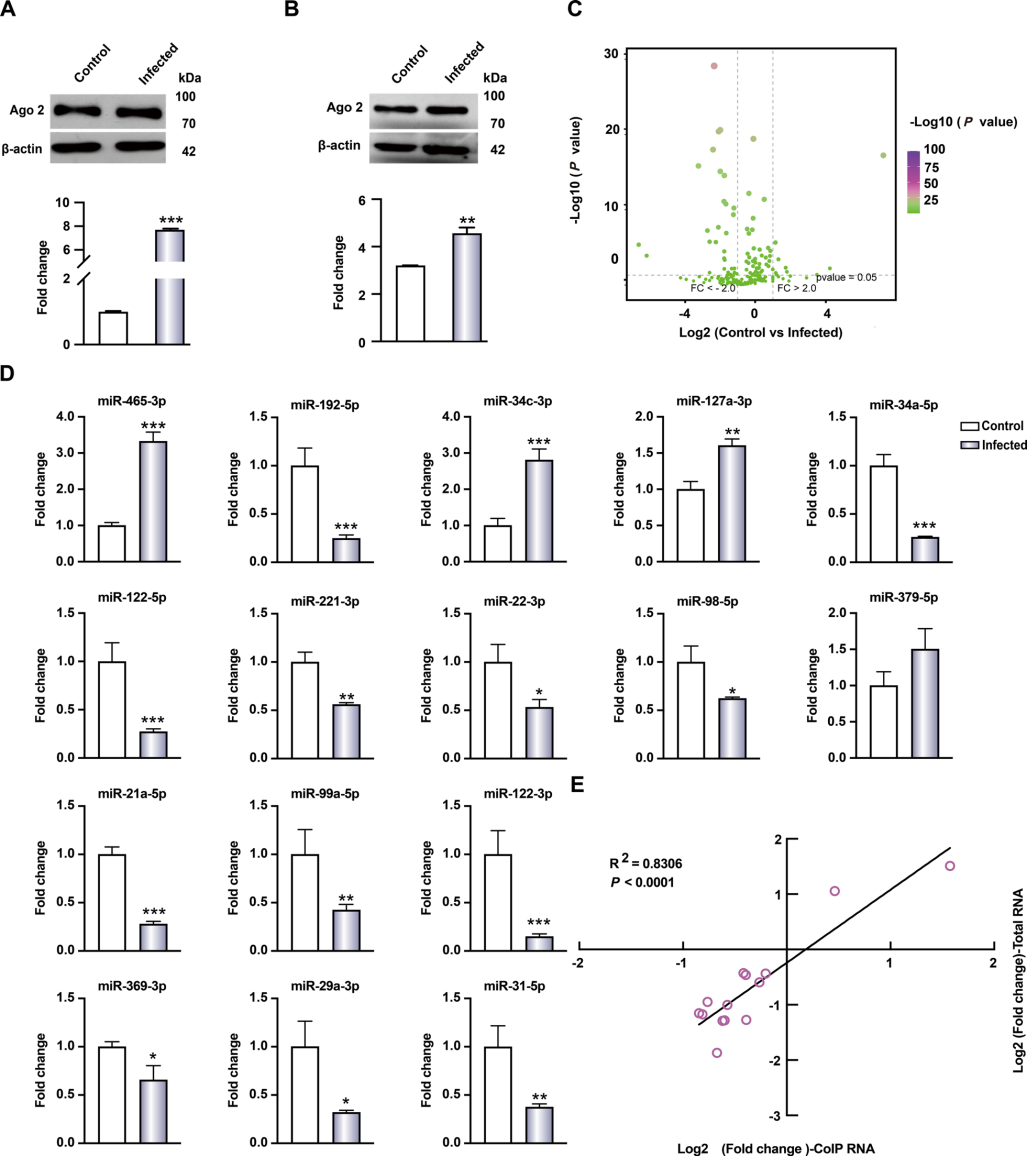
Identification of differentially expressed abmiRNAs in the hepatocytes in response to *Echinococcosis multilocularis* infection. **A** Expression levels of Ago 2 in the hepatocytes of mice infected with *E. multilocularis*, **B** relative abundance of Ago 2 in the co-immunoprecipitation products using total proteins from the hepatocytes, **C** AbmiRNA profiles in the hepatocytes in response to *E. multilocularis* infection, **D** validation of differentially expressed abmiRNAs by reverse transcription-quantitative PCR, **E** linear correlation analysis of the abundance of these abmiRNAs in total Ago 2-binding RNA and total RNA of the hepatocytes. Asterisks indicate significant difference at *P < 0.05, **P < 0.01 and ***P < 0.001. Ago 2, Agonaute 2; abmiRNAs, Ago 2-binding microRNAs

To assess the effect of *E. multilocularis* infection on the abundance of abmiRNAs, we isolated pure hepatocytes from infected mice (Additional file 1: Figure. S1) and profiled their abmiRNAs using the RNA sequencing (RNA-seq) technique. The abundance of Argonaute 2 in hepatocytes was significantly elevated in response to *E. multilocularis* infection (*P* < 0.001; Fig. 1a). This was consistent with the immunoprecipitation results, confirming that the levels of Argonaute 2 were higher in the hepatocytes from infected mice than in those of the controls (*P* < 0.001; Fig. 1b).

A total of 398 abmiRNAs were identified in both the *E. multilocularis*-infected and -uninfected groups, including 358 known and 40 novel miRNAs (Fig. 1c; Additional file 1: Table S2). Among these 398 abmiRNAs, 28 showed significant differences in abundance, with 25 showing decreased abundance and three showing increased abundance (Additional file 1:Table S3). To validate the sequencing data using enriched Argonaute 2-binding RNA by qPCR, we randomly selected 16 differentially expressed abmiRNAs and found that—with the exception of miR-379a-3p and miR-127-3p—they showed similar abundance patterns (Fig. 1d). During *E. multilocularis* infection, the abundance of the majority (25/28) of differentially expressed abmiRNAs decreased despite Argonaute 2 upregulation. We propose that this decrease results from their transcriptional downregulation. To test this possibility, we assessed the basal levels of these abmiRNAs in purified hepatocytes during the infection and confirmed that they exhibited similar expression patterns, with a significant correlation (Fig. 1e; Additional file 1: Figure S2). Taken together, these results suggest that abmiRNA abundance largely depends on the basal expression level of the specific abmiRNA.

### Circulating abmiRNA levels reflect AE disease status

**Fig. 2:**
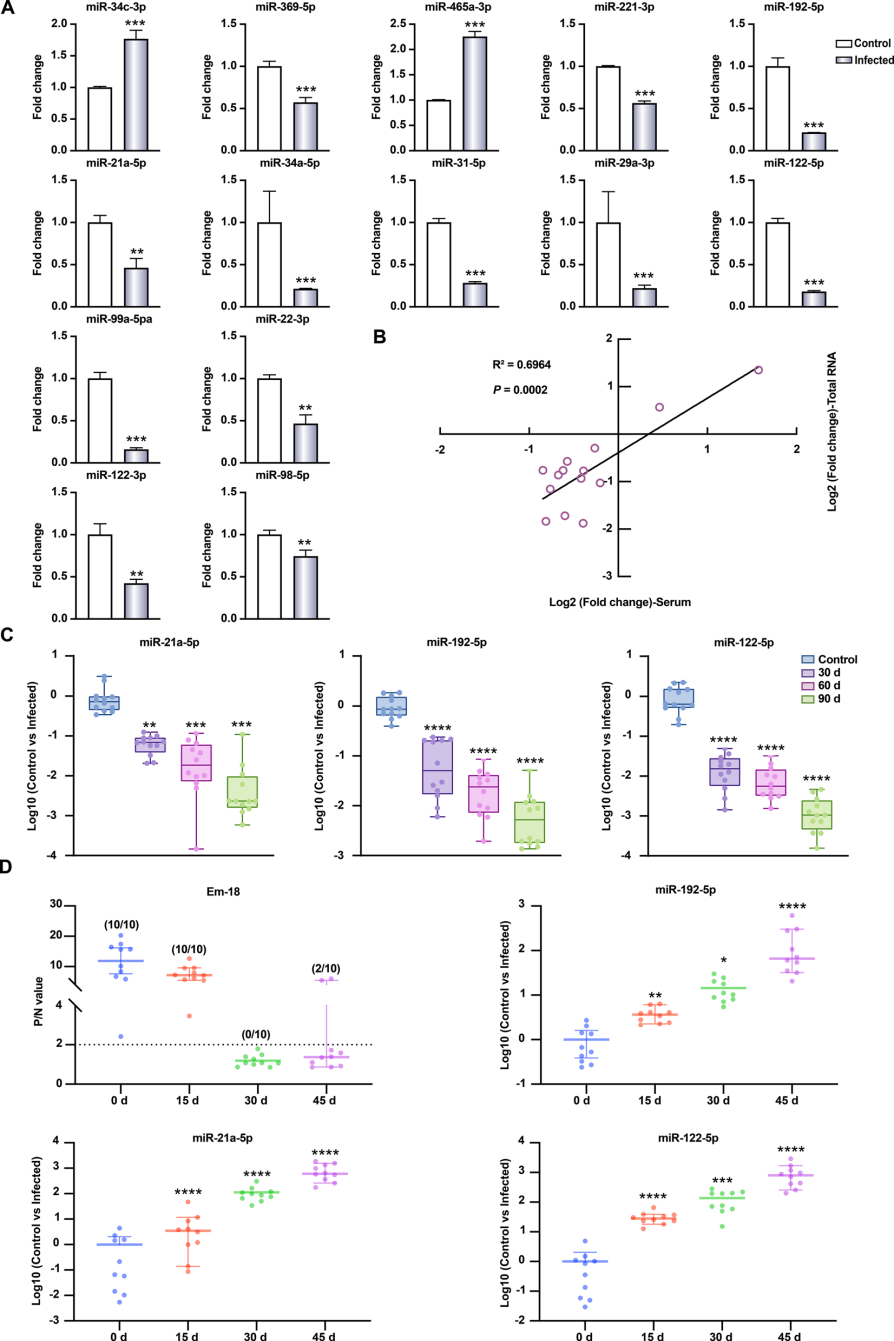
Dynamic changes in the levels of abmiRNAs circulating in the serum associated with the viability of *Echinococcosis multilocularis.* **A** Abundance of differentially expressed abmiRNAs circulating in the sera of infected mice by reverse transcription-quantitative PCR, **B** linear correlation analysis of the abundance of these abmiRNAs in the sera and total RNA of the hepatocytes, **C** serum levels of these miRNAs during *E. multilocularis* infection, **D** serum levels of these miRNAs at the indicated days after oral administration of albendazole. In** D**, for the Em-18-based enzyme-linked immunosorbent assay, the cutoff for the ratio of the optical density (OD) of the test serum (P)/OD of the negative serum (N) was set > 2 (horizontal dotted line); the numbers in parentheses are the number of positive sera/total number of sera tested. Asterisks indicate significant difference at *P < 0.05, **P < 0.01, ***P < 0.001 and ****P < 0.0001. AbmiRNAs, Ago 2-binding microRNAs

Since miRNAs can be released from organs and circulate in various forms in body fluids, we examined the abundance of differentially expressed abmiRNAs in the sera of infected mice by qPCR and found their levels were significantly positively correlated with those in the hepatocytes of infected mice (*P* < 0.0002; Fig. 2a, b).

Known liver-specific or -enriched miRNAs, such as miR-122-5p and miR-192-5p [27], were detected in the blood of infected mice at decreased levels (Fig. 2a), suggesting these circulating abmiRNAs could serve as potential biomarkers for infection monitoring. To test this possibility, we first selected nine miRNA candidates based on the following criteria: (i) no isomiRNAs with high nucleotide similarity; (ii) read counts > 100 in either control or infected groups; and (iii) two Ct values < 34 in the qPCR using a pair of negative and positive sera (Additional file 1: Table S3). qPCR analysis confirmed that, similar to known liver-specific or -enriched miR-122-5p and miR-192-5p, only one of the remaining seven miRNA candidates selected, miR-21a-5p, also showed predominant expression in the liver of healthy mice (Additional file 1: Figure S3). We therefore focused on three abmiRNAs (miR-192-5p, miR-122-5p and miR-21a-5p) for further analysis. Notably, while these miRNAs are upregulated in liver cancer and hepatitis virus infections [28, 29], all three showed significant downregulation in the sera of mice infected with *E. multilocularis* (Fig. 2c); conversely, their levels gradually increased following parasite clearance by anthelmintic treatment. These circulating miRNAs showed significant upregulation as early as 15 days post-treatment, whereas the specific antibodies against Em-18, a parasite viability marker [21], did not decrease significantly until 30 days post-treatment (Fig. 2d). These findings suggest this miRNA panel is more sensitive to parasite viability and dynamically tracks AE disease status.

### Circulating miRNA-targeted qPCR approaches demonstrate high performance

**Fig. 3:**
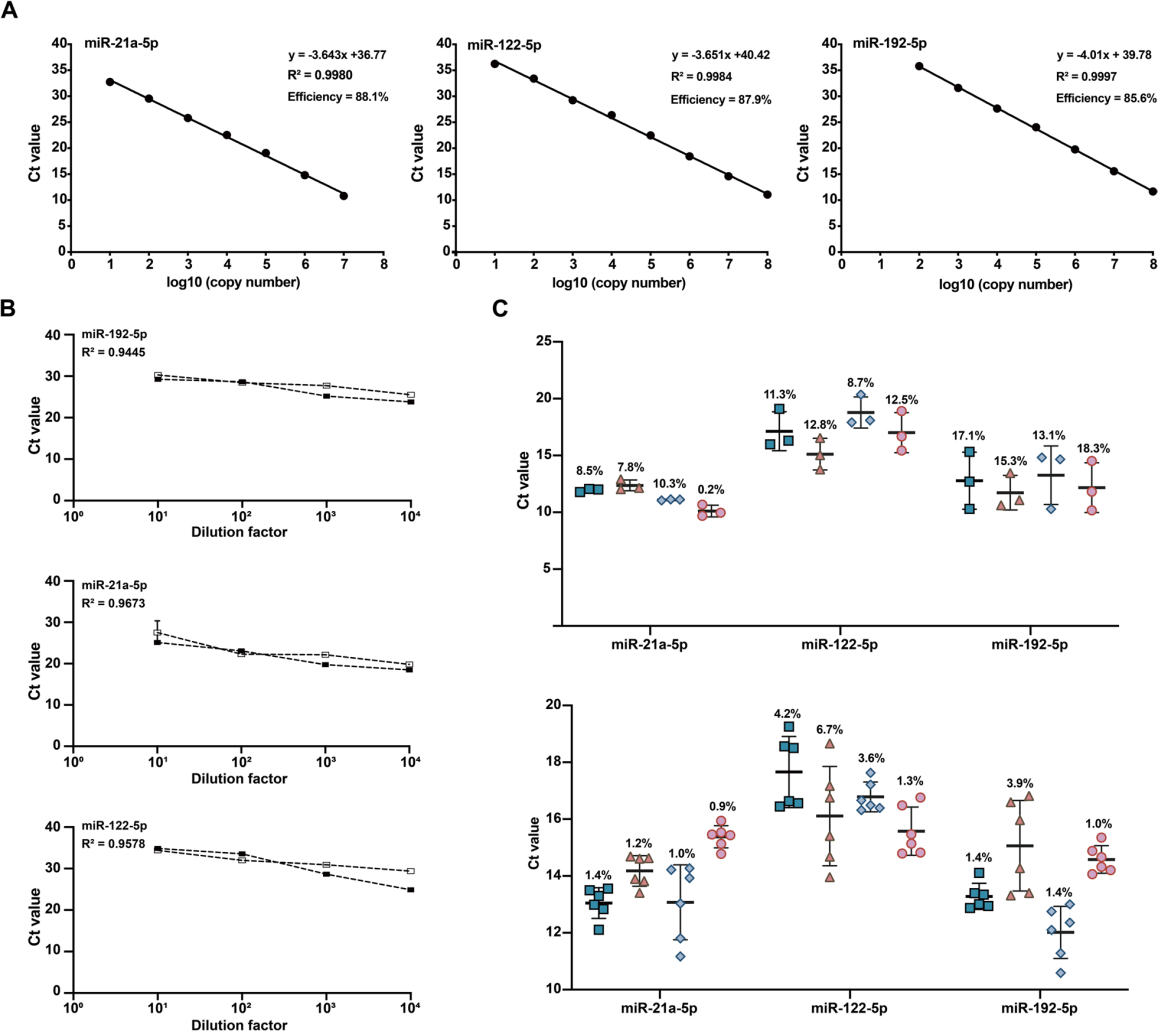
Performance of the circulating miRNA-targeted quantitative (qPCR) approaches. **A** Efficiency of the circulating miRNA-targeted qPCR approaches, **B** linear correlation analysis of the circulating miRNA-targeted qPCR approaches using two alveolar echinococcosis (AE)-positive sera serially diluted by AE-negative serum, **C** inter-assay (up) and intra-assay (below) precision analyses of the circulating miRNA-targeted qPCR approaches. Ct, Cycle threshold; miRNA, microRNA

To develop qPCR-based detection approaches, we first analyzed the amplification characteristics of the circulating miRNA panel using serially diluted miRNA-containing plasmids. The results showed acceptable amplification efficiency for individual qPCR assays, ranging from 85.6% to 88.9% (Fig. 3a). Using serial dilutions of AE-positive sera diluted by AE-negative serum, we further demonstrated that each qPCR assay exhibited efficient amplification with a strong linear correlation (*R*^2^ > 0.94; Fig. 3b).

We next evaluated the reproducibility of the PCR assays. The limits of detection were 100 copies/μl for miR-192-5p and 10 copies/μl for both miR-122-5p and miR-21a-5p, with a low coefficient of variation (CV) from 0.06% to 1.5% (Additional file 1: Table S4). Intra-assay precision analyses showed CVs < 7% (lower panel, Fig. 3c), while inter-assay precision ranged from 0.2% to 10.3% for miR-21-5p, from 8.7% to 12.8% for miR-122-5P and from 13.1% to 18.3% for miR-192-5p (upper panel, Fig. 3c). These results demonstrate that the miRNA-targeted qPCR assays perform well in terms of both amplification and reproducibility.

### qPCR detection approaches demonstrate high sensitivity and specificity

**Fig. 4:**
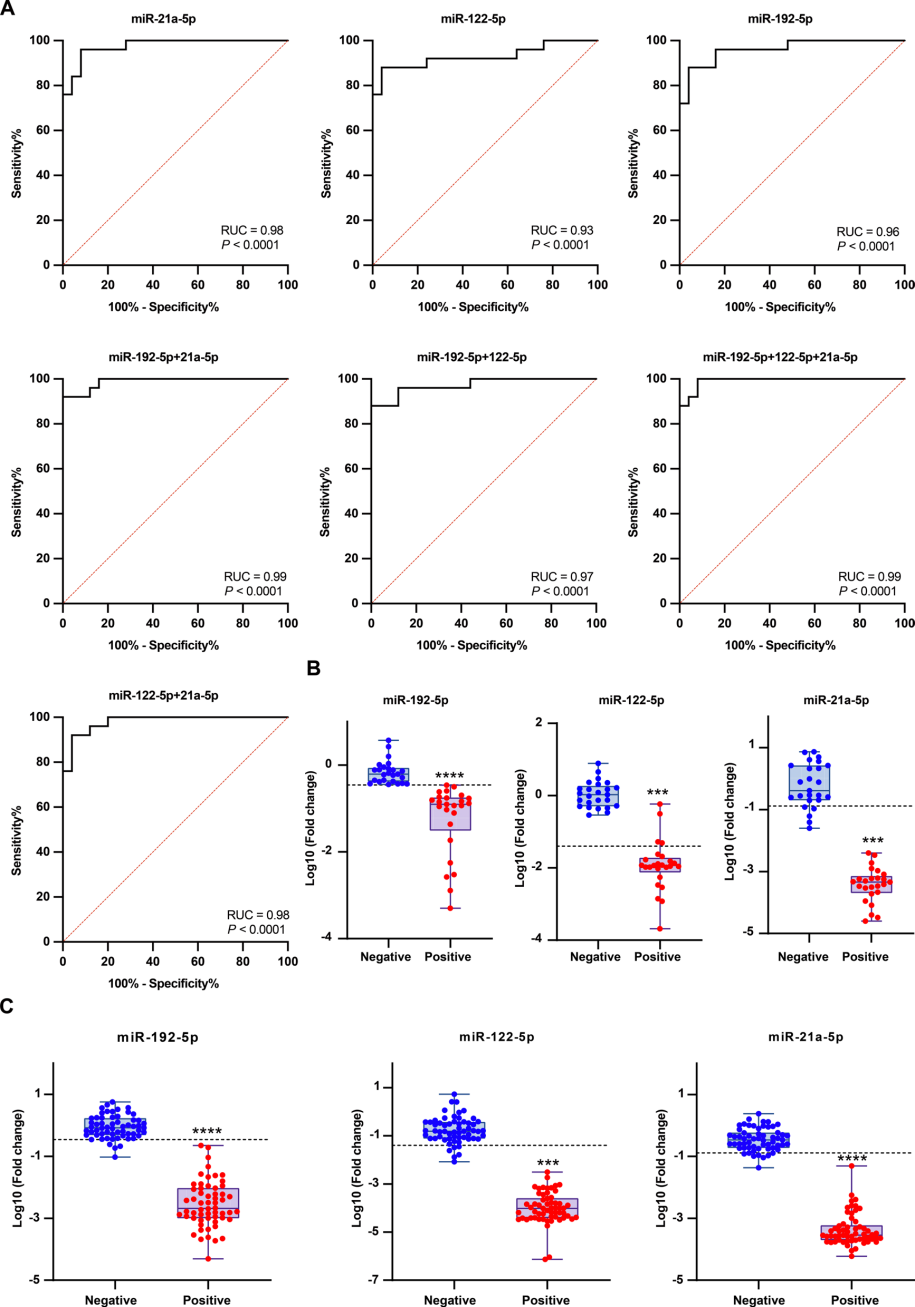
Receiver operating curve (ROC) analysis and validation of the circulating miRNA-targeted quantitative (qPCR) approaches. **A** ROC curve analysis of the qPCR approaches, **B** validation of the qPCR approaches using 25 alveolar echinococcosis (AE)-negative and 25 AE-positive blinded mouse sera, **C** validation of the qPCR approaches using 59 healthy donors and 58 echinococcosis patients blinded sera. The cutoff value for individual miRNA-targeted qPCR is shown as a dotted line. Asterisk indicate a significant difference at ***P < 0.001 and ****P < 0.0001. miR-, MicroRNA-

Using 25 AE-negative and 25 AE-positive mouse sera, we established qPCR detection approaches targeting the circulating miRNAs, setting cutoff values (when Youden’s index was maximal) at: 0.35 for miR-192-5p, 0.04 for miR-122-5p and 0.13 for miR-21a-5p (Additional file 1: Tables S5–S7). All of the miRNAs tested and their combinations showed area under the ROC curve (AUC) ≥ 0.93, with a peak AUC value of 0.99 for the combinations of miR-192-5p + miR-21a-5p (95% CI 0.97–1.00) and miR-192-5p + miR-122-5p + miR-21a-5p (95% CI 0.98–1.00) (Table 1; Fig. 4a).

**Table 1:** Receiver operating curve analysis of the quantitative PCR approaches used to detect circulating microRNA targets

Target	AUC (95% CI)	*P* value
miR-192-5p	0.96 (0.91–1.00)	< 0.0001
miR-122-5p	0.93 (0.85–1.00)	< 0.0001
miR-21a-5p	0.98 (0.94–1.00)	< 0.0001
miR-192-5p + miR-122-5p	0.97 (0.93–1.00)	< 0.0001
miR-192-5p + miR-21a-5p	0.99 (0.97–1.00)	< 0.0001
miR-122-5p + miR-21a-5p	0.98 (0.95–1.00)	< 0.0001
miR-192-5p + miR-122-5p + miR-21a-5p	0.99 (0.98–1.00)	< 0.0001

*AUC* Area under the receiver operating curve,* CI* confidence interval,* miR-* micro RNA-

To evaluate the diagnostic performance of established qPCR approaches, we analyzed 50 blinded mouse serum samples (25 AE-negative/25 AE-positive serum samples; randomly numbered with operator blinded). With the exception of miRNA-122-5p and miR-21a-5p, all analyses achieved perfect discrimination (Table 2; Fig. 4b) although there was no statistical difference between the approaches (Additional file 1: Table S8). We further compared these approaches with Em-18-based ELISA using samples from anthelmintic-treated AE mice. All qPCR methods showed comparable performance at 0, 30 and 45 days post-treatment; however, only miR-122-5p and its combination showed a significant difference at 15 days post-treatment (Table 3).

**Table 2:** Performance of the established quantitative PCR analyses for detecting target microRNAs in blinded mouse serum samples

Target	Negative rate (%)	Positive rate (%)
miR-192-5p	100 (25/25)	100 (25/25)
miR-122-5p	100 (25/25)	84 (21/25)
miR-21a-5p	80 (20/25)	100 (25/25)
miR-192-5p + miR-122-5p	100 (25/25)	100 (25/25)
miR-192-5p + miR-21a-5p	100 (25/25)	100 (25/25)
miR-122-5p + miR-21a-5p	100 (25/25)	100 (25/25)
miR-192-5p + miR-122-5p + miR-21a-5p	100 (25/25)	100 (25/25)

Alveolar echinococcosis (AE)-negative serum samples (*n* = 25) and AE-positive sera samples (*n* = 25) were included in this analysis. All samples were randomly numbered and the operator did not know which one was negative or positive

* miR-* microRNA-

**Table 3:** Comparative analysis of the established quantitative PCR analyses for detecting target microRNAs and Em-18-based enzyme-linked immunosorbent assay for the detection of AE mice following anthelmintic treatment

qPCR targeting circulating miRNAs		Em-18-based ELISA							
		0 days post-treatment^a^		15 days post-treatment		30 days post-treatment		45 days post-treatment	
		Negative	Positive	Negative	Positive	Negative	Positive	Negative	Positive
miR-192-5p	Negative	0	0	0	0	7	0	8	2
	Positive	0	10	0	10	3	0	0	0
		*P* > 0.9999		*P* > 0.9999		*P* = 0.2105		*P* = 0.4737	
miR-122-5p	Negative	0	0	10	0	10	0	8	2
	Positive	0	10	0	0	0	0	0	0
		*P* > 0.9999		*P* < 0.0001		*P* > 0.9999		*P* = 0.4737	
miR-21a-5p	Negative	0	0	0	0	10	0	8	2
	Positive	0	10	0	10	0	0	0	0
		*P* > 0.9999		*P* > 0.9999		*P* > 0.9999		*P* = 0.4737	
miR-192-5p + miR-122-5p	Negative	0	0	10	0	10	0	8	2
	Positive	0	10	0	0	0	0	0	0
		*P* > 0.9999		*P* < 0.0001		*P* > 0.9999		*P* = 0.4737	
miR-192-5p + miR-21a-5p	Negative	0	0	0	0	10	0	8	2
	Positive	0	10	0	10	0	0	0	0
		*P* > 0.9999		*P* > 0.9999		*P* > 0.9999		*P* = 0.4737	
miR-122-5p + miR-21a-5p	Negative	0	0	10	0	10	0	8	2
	Positive	0	10	0	0	0	0	0	0
		*P* > 0.9999		*P* < 0.0001		*P* > 0.9999		*P* = 0.4737	
miR-192-5p + miR-122-5p + miR-21a-5p	Negative	0	0	10	0	10	0	8	2
	Positive	0	10	0	0	0	0	0	0
		*P* > 0.9999		*P* < 0.0001		*P* > 0.9999		*P* = 0.4737	

*ELISA* Enzyme-linked immunosorbent assay,* miRNA/miR-* microRNA,* qPCR* quantitative PCR

^a^Ten infected mice were orally administrated with albendazole (200 mg/kg) and sera were collected at indicated days after treatment. The difference between every miRNA-targeting qCPR and ELISA was analyzed using Fisher’s exact test

These results demonstrate that circulating miR-192-5p, miR-122-5p and miR-21-5p serve as reliable biomarkers, with established qPCR approaches showing good sensitivity and specificity.

### The miR-192-5p and miR-122-5p combination serves as a reliable diagnostic signature for echinococcosis

To validate the diagnostic reliability of the three circulating miRNAs and their combinations, we analyzed 107 blinded human serum samples (59 from healthy donors and 58 from echinococcosis patients; randomly numbered with operator blinded). The results showed that, for 59 negative control sera, only two combinations, miR-192-5p + miR-122-5p and miR-192-5p + miR-122-5p + miR-21a-5p, had a negative rate of 100%. However, for 58 AE-positive sera, all miRNAs and combinations had a positive rate of 100% (Table 4; Fig. 4c). These results demonstrate that the two-miRNA panel (miR-192-5p + miR-122-5p) provides optimal diagnostic precision for echinococcosis.

**Table 4:** Performance of the established quantitative PCR approaches for detecting echinococcosis in blinded human serum samples

Target	Negative rate (%)	Positive rate (%)
miR-192-5p	91.5 (54/59)	100 (58/58)
miR-122-5p	89.8 (53/59)	100 (58/58)
miR-21a-5p	86.4 (51/59)	100 (58/58)
miR-192-5p + miR-122-5p	100 (59/59)	100 (58/58)
miR-192-5p + miR-21a-5p	96.6 (57/59)	100 (58/58)
miR-122-5p + miR-21a-5p	98.3 (58/59)	100 (58/58)
miR-192-5p + miR-122-5p + miR-21a-5p	100 (59/59)	100 (58/58)

Serum samples were collected from 59 healthy people and 58 patients with echinococcosis and analyzed. All samples were randomly numbered and the operator did not know which one was negative or positive

* miR-* MicroRNA

## Discussion

Among the major food-borne parasitic diseases, echinococcosis contributes to a relatively high global disease burden [30]. Accurate diagnosis is critical for effective disease control, yet this remains challenging due to the limitations of current diagnostic methods [31]. Growing evidence demonstrates that dysregulated host miRNAs are closely associated with parasitic disease pathogenesis [32]. Moreover, miRNAs released into the circulation from various tissues present promising non-invasive biomarkers [33, 34]. Our study identified three liver-enriched circulating miRNAs whose levels are correlated with the parasite viability. qPCR detection of these miRNAs, particularly the miR-122-5p + miR-192-5p combination, showed excellent sensitivity and specificity in both animal models and human patients. Notably, these miRNA markers demonstrated greater sensitivity to parasite viability than the established Em-18 antigen test. While the utility of these miRNA markers in post-treatment monitoring requires further validation (due to limited sera from cured patients in our study), these findings strongly support their diagnostic and potential prognostic value.

The invasive growth pattern of *Echinococcus* species often leads to misdiagnosis as liver tumor, and vice versa in some cases [35, 36], highlighting the need for reliable differentiation. Interestingly, while miR-122-5p, miR-21-5p and miR-192-5p serve as established liver cancer biomarkers [37, 38] and facilitate clinicians in distinguishing hepatocellular carcinoma (HCC) from cirrhosis [28, 29], they exhibit opposing trends in AE: whereas the levels of these miRNAs increase in HCC and HBeAg-positive hepatitis [28, 29], they decline progressively during *E. multilocularis* infection. At present, we have no reliable explanations for the downregulation of these predominant miRNAs, although one possibility is that such downregulation may be attributed to, at least partially, parasite manipulation, possibly through the release of excreted/secreted (E/S) products and extracellular vesicles [39, 40]. However, this inverse expression pattern enables echinococcosis to be clearly distinguished from both liver cancer and hepatitis B virus infection using these miRNA signatures. Nevertheless, the diagnostic efficacy must be further evaluated, especially in early diagnosis. Of great interest is the standardization of the reagents and operation protocols of these established qPCR approaches as well as multi-center validation in future studies.

Early diagnosis is crucial for effective echinococcosis control, given its long latent period and limited treatment options for advanced cases [41]. Currently, no precise early diagnostic methods exist. Recent approaches combining vibrational spectroscopy with machine learning achieved > 93% diagnostic accuracy in mouse sera [42]. Similarly, in one study, both circulating parasite proteins, thioredoxin peroxidase 1 and transitional endoplasmic reticulum ATPase enabled AE detection as early as 10 days post-infection [21]. However, the clinical utility of these approaches for early human diagnosis requires further validation. Our study demonstrates that three liver-enriched miRNAs, particularly the miR-122-5p + miR-192-5p combination, serve as precise serum biomarkers for AE detection from 30 days post-infection onward. Their potential for early diagnosis could not be evaluated in the present study due to the lack of early-stage human samples.

Albendazole and mebendazole, while effective against *E. multilocularis* larvae, exhibit toxicity [8], making treatment monitoring essential for these long-term therapies. Although Em18-based ELISA remains the gold standard for assessing parasite viability [43], our findings reveal that miR-122-5p-targeted qPCR outperforms Em18-based ELISA in terms of sensitivity, detecting treatment effects as early as 15 days post-treatment. This aligns with albendazole’s known parasiticidal effects within 7 days of treatment [44]. These results position the miR-122-5p + miR-192-5p combination as a promising tool for treatment monitoring. It should be noted that although in the present these circulating miRNA levels strongly correlated with infection status within treatment periods, we were unable to provide long-term follow-up data for prognosis. A worthwhile future research goal would be to evaluate the diagnostic values of the miR-122-5p + miR-192-5p combination in prognosis management.

In conclusion, serum miR-122-5p and miR-192-5p levels dynamically reflect *E. multilocularis* infection status, offering both diagnostic and potential prognostic value for echinococcosis management.

## Supplementary Information

Additional file 1. Fig. S1 Analysis of the isolated hepatocytes by RT-qPCR. Additional file 1. Fig. S2 Comparative analysis of the basal levels of differentially expressed abmiRNAs in the hepatocytes of infected mice. Additional file 1. Fig. S3 Analysis of the relative expression levels of abmiRNAs in the organs/tissues of healthy mice. Additional file 1. Table S1 The primers used in this study. Additional file 1. Table S2 Argonaute 2-binding miRNAs (abmiRNAs) in the hepatocytes of mice with or without Echinococcus multilocularis infection. Additional file 1. Table S3 Differentially expressed abmiRNAs in response to Echinococcus multilocularis infection. Additional file 1. Table S4 Analysis of the sensitivity of circulating miRNA-targeted qPCR using recombinant plasmids. Additional file 1. Additional file 1. Table S5 Youden’s index analysis of miR-192-5p-targeted qPCR. Additional file 1. Table S6 Youden’s index analysis of miR-122-5p-targeted qPCR. Additional file 1. Table S7 Youden’s index analysis of miR-21a-5p-targeted qPCR. Additional file 1. Table S8 Comparison of the established qPCR approaches targeting individual circulating miRNAs molecules and their combinations in discriminating E. multilocularis-infected mice from healthy mice.

## Data Availability

All study data are included in the article and supplementary files.
