# Supplementary material for: Circulating microRNA signatures for echinococcosis
Source: Parasit Vectors. 2025 Dec 1;19:10. doi: 10.1186/s13071-025-07161-8 (PMC12777240; doi:10.1186/s13071-025-07161-8)
Supplement: Supplementary file 1 — Additional file 1. Fig. S1 Analysis of the isolated hepatocytes by RT-qPCR. Additional file 1. Fig. S2 Comparative analysis of the basal levels of differentially expressed abmiRNAs in the hepatocytes of infected mice. Additional file 1. Fig. S3 Analysis of the relative expression levels of abmiRNAs in the organs/tissues of healthy mice. Additional file 1. Table S1 The primers used in this study. Additional file 1. Table S2 Argonaute 2-binding miRNAs (abmiRNAs) in the hepatocytes of mice with or without Echinococcus multilocularis infection. Additional file 1. Table S3 Differentially expressed abmiRNAs in response to Echinococcus multilocularis infection. Additional file 1. Table S4 Analysis of the sensitivity of circulating miRNA-targeted qPCR using recombinant plasmids. Additional file 1. Additional file 1. Table S5 Youden’s index analysis of miR-192-5p-targeted qPCR. Additional file 1. Table S6 Youden’s index analysis of miR-122-5p-targeted qPCR. Additional file 1. Table S7 Youden’s index analysis of miR-21a-5p-targeted qPCR. Additional file 1. Table S8 Comparison of the established qPCR approaches targeting individual circulating miRNAs molecules and their combinations in discriminating E. multilocularis-infected mice from healthy mice. [file 13071_2025_7161_MOESM1_ESM.docx]

**Supplementary file**


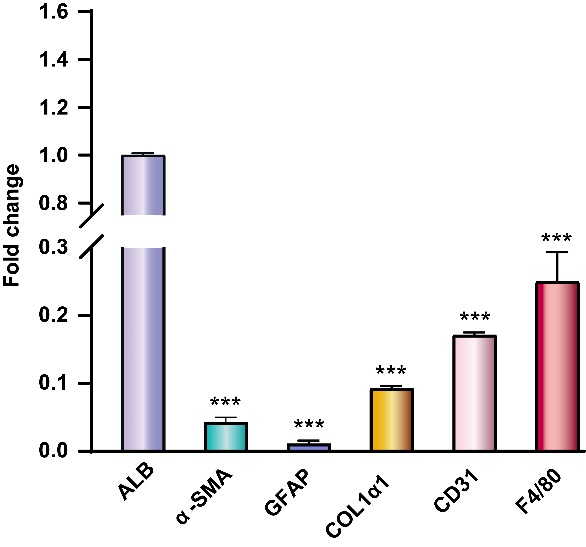


Fig. S1 Analysis of the isolated hepatocytes by RT-qPCR

The purity of the isolated hepatocytes was analyzed using specific markers. ALB, albumin, a marker for hepatocytes; α-SMA, α-smooth muscle actin, a marker for activated hepatic stellate cells; GFAP, glial fibrillary acidic protein, a marker for quiescent hepatic stellate cells; COL1α1, collagen type I α1, a marker for activated hepatic stellate cells; CD31, a marker for endothelial cells: F4/80, a marker for Kupffer cells. Data are shown as mean ± SD (n = 6). ‘***’, *P* < 0.001 (ALB group vs any group of the rest).


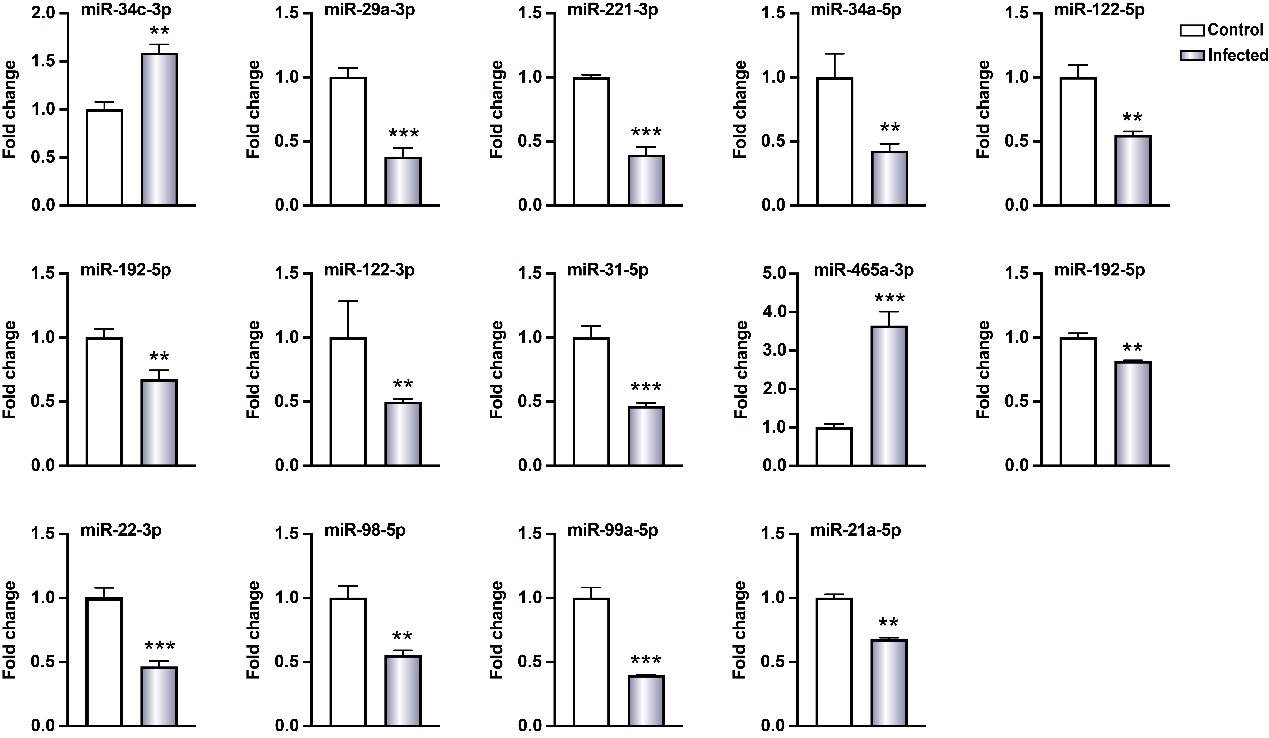


Fig. S2 Comparative analysis of the basal levels of differentially expressed abmiRNAs in the hepatocytes of infected mice

The hepatocytes were isolated from the liver of mice infected with *E. multilocularis*, followed by the extraction of total RNA. cDNA was synthesized and then qPCR was immediately conducted. The relative expression levels of the differentially expressed abmiRNAs were calculated using the formula 2^-ΔΔCt^ and cel-miR-39-3p was served as a reference. Each sample was set in triplicate. AbmiRNAs, Argonaute 2-binding miRNAs. Data are shown as mean ± SD (n = 3). ‘**’, *P* < 0.01; ‘***’, *P* < 0.001.


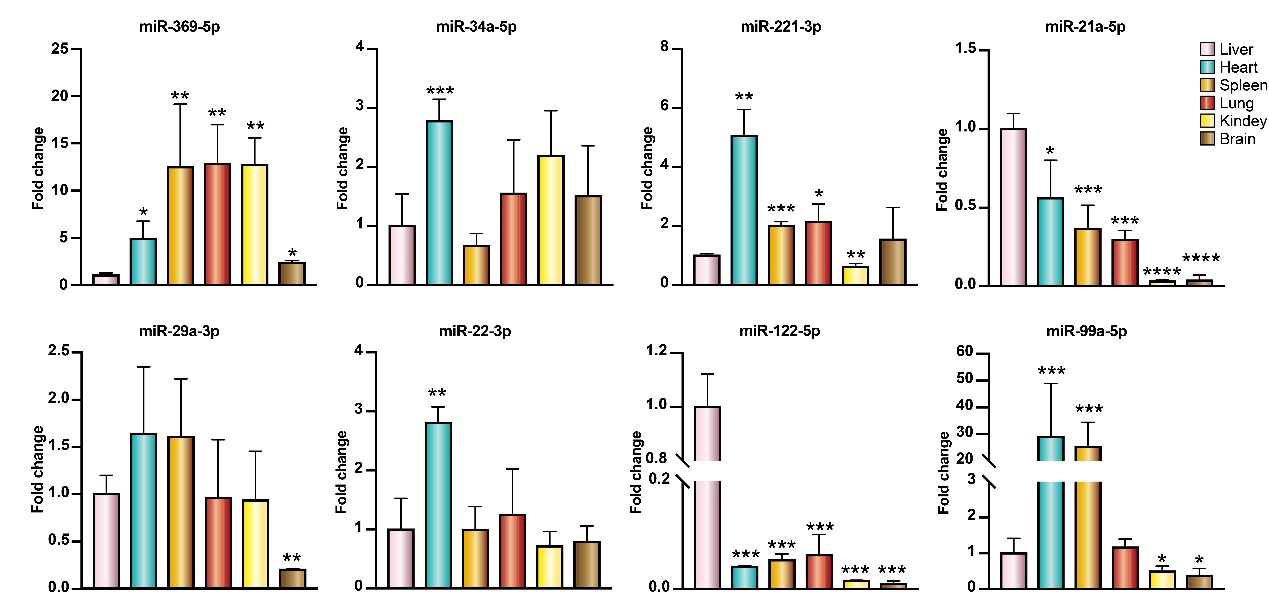


Fig. S3 Analysis of the relative expression levels of abmiRNAs in the organs/tissues of healthy mice

The main organs/tissues of healthy mice were dissected, followed by the extraction of total RNA. cDNA was synthesized and then qPCR was immediately conducted. The relative expression levels of abmiRNAs were calculated using the formula 2^-ΔΔCt^ and U6 was utilized as a reference. Each sample was set in triplicate. AbmiRNAs, Argonaute 2-binding miRNAs. Data are shown as mean ± SD (n = 3). ‘*’, *P* < 0.05; ‘**’, *P* < 0.01; ‘***’, *P* < 0.001; ‘****’, *P* < 0.0001 (Liver group vs any group of the rest).

Table S1 The primers used in this study

| Primer | Sequence (5’-3’) |
| --- | --- |
| miR-122-5p | GAGTGTGACAATGGTGTTTGAAA |
| miR-192-5p | CTGACCTATGAATTGACAGCCAAA |
| miR-21a-5p | TAGCTTATCAGACTGATGTTGAAAA |
| miR-29a-3p | CACCATCTGAAATCGGTTAA |
| miR-99a-5p | CGTAGATCCGATCTTGTGAA |
| miR-127-3p | GATCCGTCTGAGCTTGGCTAAA |
| miR-22-3p | AAGCTGCCAGTTGAAGAACTGTAA |
| miR-221-3p | TACATTGTCTGCTGGGTTTCAAA |
| miR-31-5p | GCAAGATGCTGGCATAGCTGAA |
| miR-34a-5p | GCAGTGTCTTAGCTGGTTGTAAA |
| miR-34c-3p | ACTAACCACACAGCCAGGAAA |
| miR-369-3p | CAATAATACATGGTTGATCTTTAAAA |
| miR-379-5p | TGGTAGACTATGGAACGTAGGAAAA |
| miR-465c-3p | GATCAGGGCCTTTCTAAGTAGAAA |
| miR-98-5p | TGAGGTAGTAAGTTGTATTGTTAAAAA |
| miR-122-3p | GCAAACGCCATTATCACACTAAA |
| RT primer | GCGAGCACAGAATTAATACGACTCACTATAGG(T)_12_VN^1^ |
| Universal reverse primer | GCGAGCACAGAATTAATACGAC |

^1^‘V' stands for A, G or C; ‘N' stands for A, T, G or C.

Table S2 Argonaute 2-binding miRNAs (abmiRNAs) in the hepatocytes of mice with or without *Echinococcus multilocularis* infection

| miRNA | Read counts in the control | Read counts in the infected group | Fold change | *P* value |
| --- | --- | --- | --- | --- |
| miR-1197-3p | 2 | 1 | 0.50 | 0.98856 |
| miR-122b-3p | 2 | 1 | 0.50 | 0.98856 |
| miR-140-3p | 2 | 1 | 0.50 | 0.98856 |
| miR-195a-5p | 2 | 1 | 0.50 | 0.98856 |
| miR-20b-5p | 2 | 1 | 0.50 | 0.98856 |
| miR-3068-5p | 2 | 1 | 0.50 | 0.98856 |
| miR-376c-5p | 2 | 1 | 0.50 | 0.98856 |
| miR-378b | 2 | 1 | 0.50 | 0.98856 |
| miR-654-3p | 2 | 1 | 0.50 | 0.98856 |
| miR-874-5p | 2 | 1 | 0.50 | 0.98856 |
| novel-miR-220-5p | 2 | 1 | 0.50 | 0.98856 |
| miR-486b-3p | 7 | 4 | 0.57 | 0.977168 |
| miR-6538 | 5 | 3 | 0.60 | 0.946276 |
| miR-151-5p | 181 | 113 | 0.62 | 0.931814 |
| miR-218-5p | 35 | 22 | 0.62 | 0.92332 |
| miR-103-3p | 27 | 17 | 0.63 | 0.91982 |
| miR-99b-3p | 8 | 5 | 0.62 | 0.91906 |
| miR-335-5p | 9 | 5 | 0.55 | 0.91726 |
| miR-3965 | 4 | 2 | 0.50 | 0.917236 |
| miR-10a-5p | 181 | 114 | 0.63 | 0.873534 |
| miR-7a-5p | 41 | 24 | 0.58 | 0.858672 |
| miR-497a-5p | 6 | 3 | 0.50 | 0.854362 |
| miR-139-5p | 20 | 13 | 0.65 | 0.850618 |
| miR-185-5p | 34 | 22 | 0.64 | 0.840806 |
| miR-145a-3p | 3 | 2 | 0.66 | 0.83868 |
| miR-181d-5p | 3 | 2 | 0.66 | 0.83868 |
| miR-1843a-5p | 3 | 2 | 0.66 | 0.83868 |
| miR-669o-5p | 3 | 2 | 0.66 | 0.83868 |
| miR-871-3p | 3 | 2 | 0.66 | 0.83868 |
| miR-877-5p | 3 | 2 | 0.66 | 0.83868 |
| novel-miR-432-5p | 3 | 2 | 0.66 | 0.83868 |
| miR-181a-1-3p | 6 | 4 | 0.66 | 0.837852 |
| miR-1983 | 6 | 4 | 0.66 | 0.837852 |
| miR-322-5p | 6 | 4 | 0.66 | 0.837852 |
| miR-350-3p | 6 | 4 | 0.66 | 0.837852 |
| miR-425-3p | 6 | 4 | 0.66 | 0.837852 |
| miR-377-3p | 20 | 11 | 0.55 | 0.799118 |
| miR-140-5p | 24 | 16 | 0.66 | 0.783626 |
| let-7i-3p | 1 | 0 | 0.26 | 0.76272 |
| miR-101b-5p | 1 | 0 | 0.26 | 0.76272 |
| miR-103-1-5p | 1 | 0 | 0.26 | 0.76272 |
| miR-107-3p | 1 | 0 | 0.26 | 0.76272 |
| miR-133a-3p | 1 | 0 | 0.26 | 0.76272 |
| miR-134-3p | 1 | 0 | 0.26 | 0.76272 |
| miR-148a-5p | 1 | 0 | 0.26 | 0.76272 |
| miR-148b-5p | 1 | 0 | 0.26 | 0.76272 |
| miR-153-3p | 1 | 0 | 0.26 | 0.76272 |
| miR-17-3p | 1 | 0 | 0.26 | 0.76272 |
| miR-187-3p | 1 | 0 | 0.26 | 0.76272 |
| miR-190a-5p | 1 | 0 | 0.26 | 0.76272 |
| miR-194-2-3p | 1 | 0 | 0.26 | 0.76272 |
| miR-1941-3p | 1 | 0 | 0.26 | 0.76272 |
| miR-1948-3p | 1 | 0 | 0.26 | 0.76272 |
| miR-195a-3p | 1 | 0 | 0.26 | 0.76272 |
| miR-1981-3p | 1 | 0 | 0.26 | 0.76272 |
| miR-1b-5p | 1 | 0 | 0.26 | 0.76272 |
| miR-200c-5p | 1 | 0 | 0.26 | 0.76272 |
| miR-215-5p | 1 | 0 | 0.26 | 0.76272 |
| miR-216a-5p | 1 | 0 | 0.26 | 0.76272 |
| miR-21a-3p | 1 | 0 | 0.26 | 0.76272 |
| miR-222-5p | 1 | 0 | 0.26 | 0.76272 |
| miR-223-5p | 1 | 0 | 0.26 | 0.76272 |
| miR-26a-2-3p | 1 | 0 | 0.26 | 0.76272 |
| miR-297c-5p | 1 | 0 | 0.26 | 0.76272 |
| miR-29b-2-5p | 1 | 0 | 0.26 | 0.76272 |
| miR-302d-3p | 1 | 0 | 0.26 | 0.76272 |
| miR-3065-5p | 1 | 0 | 0.26 | 0.76272 |
| miR-3071-3p | 1 | 0 | 0.26 | 0.76272 |
| miR-3074-2-3p | 1 | 0 | 0.26 | 0.76272 |
| miR-3074-5p | 1 | 0 | 0.26 | 0.76272 |
| miR-30b-3p | 1 | 0 | 0.26 | 0.76272 |
| miR-32-3p | 1 | 0 | 0.26 | 0.76272 |
| miR-3470b | 1 | 0 | 0.26 | 0.76272 |
| miR-34b-5p | 1 | 0 | 0.26 | 0.76272 |
| miR-34c-5p | 1 | 0 | 0.26 | 0.76272 |
| miR-3535 | 1 | 0 | 0.26 | 0.76272 |
| miR-455-3p | 1 | 0 | 0.26 | 0.76272 |
| miR-466f-3p | 1 | 0 | 0.26 | 0.76272 |
| miR-491-5p | 1 | 0 | 0.26 | 0.76272 |
| miR-511-3p | 1 | 0 | 0.26 | 0.76272 |
| miR-542-3p | 1 | 0 | 0.26 | 0.76272 |
| miR-669l-5p | 1 | 0 | 0.26 | 0.76272 |
| miR-7219-3p | 1 | 0 | 0.26 | 0.76272 |
| miR-7240-5p | 1 | 0 | 0.26 | 0.76272 |
| miR-743a-3p | 1 | 0 | 0.26 | 0.76272 |
| novel-miR-149-5p | 1 | 0 | 0.26 | 0.76272 |
| novel-miR-186-5p | 1 | 0 | 0.26 | 0.76272 |
| novel-miR-213-3p | 1 | 0 | 0.26 | 0.76272 |
| novel-miR-2-5p | 1 | 0 | 0.26 | 0.76272 |
| novel-miR-341-3p | 1 | 0 | 0.26 | 0.76272 |
| novel-miR-365-5p | 1 | 0 | 0.26 | 0.76272 |
| novel-miR-41-3p | 1 | 0 | 0.26 | 0.76272 |
| novel-miR-421-5p | 1 | 0 | 0.26 | 0.76272 |
| novel-miR-433-5p | 1 | 0 | 0.26 | 0.76272 |
| novel-miR-474-5p | 1 | 0 | 0.26 | 0.76272 |
| let-7d-3p | 7 | 5 | 0.71 | 0.748672 |
| novel-miR-450-5p | 16 | 11 | 0.68 | 0.748364 |
| miR-375-3p | 71 | 41 | 0.57 | 0.74484 |
| miR-204-5p | 53 | 35 | 0.66 | 0.744286 |
| miR-129-5p | 3 | 1 | 0.33 | 0.735854 |
| miR-203b-5p | 3 | 1 | 0.33 | 0.735854 |
| miR-221-5p | 3 | 1 | 0.33 | 0.735854 |
| miR-27b-5p | 3 | 1 | 0.33 | 0.735854 |
| miR-30e-3p | 3 | 1 | 0.33 | 0.735854 |
| novel-miR-143-3p | 3 | 1 | 0.33 | 0.735854 |
| miR-144-5p | 4 | 3 | 0.74 | 0.731056 |
| miR-758-3p | 4 | 3 | 0.74 | 0.731056 |
| miR-193a-3p | 5 | 2 | 0.40 | 0.706212 |
| miR-24-2-5p | 5 | 2 | 0.40 | 0.706212 |
| miR-299a-5p | 5 | 2 | 0.40 | 0.706212 |
| miR-450a-5p | 5 | 2 | 0.40 | 0.706212 |
| novel-miR-214-3p | 5 | 2 | 0.40 | 0.706212 |
| miR-181c-5p | 26 | 18 | 0.69 | 0.685388 |
| miR-186-5p | 11 | 8 | 0.72 | 0.685032 |
| miR-505-3p | 8 | 6 | 0.74 | 0.67354 |
| miR-23b-3p | 399 | 238 | 0.59 | 0.655578 |
| let-7c-2-3p | 1 | 1 | 0.99 | 0.653864 |
| let-7e-3p | 1 | 1 | 0.99 | 0.653864 |
| miR-10a-3p | 1 | 1 | 0.99 | 0.653864 |
| miR-1249-3p | 1 | 1 | 0.99 | 0.653864 |
| miR-130b-3p | 1 | 1 | 0.99 | 0.653864 |
| miR-151-3p | 1 | 1 | 0.99 | 0.653864 |
| miR-155-5p | 1 | 1 | 0.99 | 0.653864 |
| miR-191-3p | 1 | 1 | 0.99 | 0.653864 |
| miR-19b-1-5p | 1 | 1 | 0.99 | 0.653864 |
| miR-28a-3p | 1 | 1 | 0.99 | 0.653864 |
| miR-409-5p | 1 | 1 | 0.99 | 0.653864 |
| miR-433-5p | 1 | 1 | 0.99 | 0.653864 |
| miR-669p-5p | 1 | 1 | 0.99 | 0.653864 |
| miR-741-5p | 1 | 1 | 0.99 | 0.653864 |
| novel-miR-100-5p | 1 | 1 | 0.99 | 0.653864 |
| novel-miR-293-5p | 1 | 1 | 0.99 | 0.653864 |
| novel-miR-402-5p | 1 | 1 | 0.99 | 0.653864 |
| novel-miR-424-5p | 1 | 1 | 0.99 | 0.653864 |
| novel-miR-474-3p | 1 | 1 | 0.99 | 0.653864 |
| miR-340-5p | 18 | 9 | 0.50 | 0.64791 |
| miR-590-3p | 12 | 9 | 0.74 | 0.625202 |
| miR-181b-5p | 99 | 56 | 0.56 | 0.605728 |
| miR-335-3p | 22 | 16 | 0.72 | 0.59566 |
| miR-872-5p | 6 | 5 | 0.83 | 0.578878 |
| miR-148b-3p | 2 | 2 | 0.99 | 0.575182 |
| novel-miR-202-3p | 2 | 2 | 0.99 | 0.575182 |
| miR-15a-5p | 53 | 37 | 0.69 | 0.557266 |
| miR-149-5p | 62 | 43 | 0.69 | 0.55096 |
| miR-1247-5p | 6 | 2 | 0.33 | 0.532456 |
| miR-136-3p | 6 | 2 | 0.33 | 0.532456 |
| miR-22-5p | 6 | 2 | 0.33 | 0.532456 |
| miR-214-3p | 8 | 3 | 0.37 | 0.52914 |
| miR-26b-3p | 4 | 1 | 0.25 | 0.52312 |
| miR-138-5p | 7 | 6 | 0.85 | 0.521486 |
| miR-9-5p | 7 | 6 | 0.85 | 0.521486 |
| miR-144-3p | 23 | 11 | 0.47 | 0.51761 |
| miR-200a-3p | 3 | 3 | 0.99 | 0.513236 |
| miR-29c-5p | 3 | 3 | 0.99 | 0.513236 |
| miR-532-3p | 3 | 3 | 0.99 | 0.513236 |
| miR-741-3p | 3 | 3 | 0.99 | 0.513236 |
| miR-199b-3p | 41 | 21 | 0.51 | 0.505078 |
| miR-361-5p | 15 | 12 | 0.79 | 0.481752 |
| miR-196b-5p | 18 | 8 | 0.44 | 0.474996 |
| miR-363-3p | 8 | 7 | 0.87 | 0.472326 |
| let-7f-2-3p | 2 | 0 | 0.13 | 0.471014 |
| miR-103-2-5p | 2 | 0 | 0.13 | 0.471014 |
| miR-125b-1-3p | 2 | 0 | 0.13 | 0.471014 |
| miR-132-5p | 2 | 0 | 0.13 | 0.471014 |
| miR-1843b-5p | 2 | 0 | 0.13 | 0.471014 |
| miR-331-3p | 2 | 0 | 0.13 | 0.471014 |
| miR-3470a | 2 | 0 | 0.13 | 0.471014 |
| miR-34a-3p | 2 | 0 | 0.13 | 0.471014 |
| miR-351-5p | 2 | 0 | 0.13 | 0.471014 |
| miR-377-5p | 2 | 0 | 0.13 | 0.471014 |
| miR-494-3p | 2 | 0 | 0.13 | 0.471014 |
| miR-7689-3p | 2 | 0 | 0.13 | 0.471014 |
| miR-98-3p | 2 | 0 | 0.13 | 0.471014 |
| novel-miR-205-5p | 2 | 0 | 0.13 | 0.471014 |
| novel-miR-24-5p | 2 | 0 | 0.13 | 0.471014 |
| novel-miR-489-3p | 2 | 0 | 0.13 | 0.471014 |
| miR-27b-3p | 295 | 170 | 0.57 | 0.462622 |
| miR-33-5p | 4 | 4 | 0.99 | 0.46203 |
| miR-26b-5p | 215 | 144 | 0.67 | 0.458682 |
| miR-381-3p | 42 | 21 | 0.50 | 0.446588 |
| miR-486b-5p | 16 | 13 | 0.81 | 0.44325 |
| miR-543-3p | 16 | 13 | 0.81 | 0.44325 |
| miR-652-3p | 39 | 29 | 0.74 | 0.438836 |
| miR-495-3p | 35 | 17 | 0.48 | 0.436496 |
| miR-455-5p | 9 | 8 | 0.88 | 0.429602 |
| miR-433-3p | 11 | 4 | 0.36 | 0.409546 |
| miR-29c-3p | 17 | 7 | 0.41 | 0.39938 |
| miR-301a-3p | 7 | 2 | 0.28 | 0.394496 |
| miR-30e-5p | 7 | 2 | 0.28 | 0.394496 |
| miR-671-5p | 7 | 2 | 0.28 | 0.394496 |
| miR-382-5p | 21 | 9 | 0.43 | 0.386196 |
| miR-423-5p | 18 | 15 | 0.83 | 0.376808 |
| miR-574-5p | 25 | 11 | 0.44 | 0.371494 |
| miR-30c-5p | 42 | 20 | 0.47 | 0.352144 |
| miR-425-5p | 26 | 21 | 0.80 | 0.349962 |
| miR-30a-5p | 80 | 41 | 0.51 | 0.336732 |
| miR-142a-5p | 1 | 2 | 1.99 | 0.31917 |
| miR-15b-3p | 1 | 2 | 1.99 | 0.31917 |
| miR-224-3p | 1 | 2 | 1.99 | 0.31917 |
| miR-30d-3p | 1 | 2 | 1.99 | 0.31917 |
| miR-381-5p | 1 | 2 | 1.99 | 0.31917 |
| miR-465b-5p | 1 | 2 | 1.99 | 0.31917 |
| miR-743a-5p | 1 | 2 | 1.99 | 0.31917 |
| miR-93-3p | 1 | 2 | 1.99 | 0.31917 |
| miR-28a-5p | 24 | 20 | 0.83 | 0.312956 |
| miR-467e-5p | 2 | 3 | 1.49 | 0.311684 |
| novel-miR-72-3p | 2 | 3 | 1.49 | 0.311684 |
| miR-378a-3p | 123 | 65 | 0.52 | 0.309792 |
| miR-324-3p | 3 | 4 | 1.32 | 0.295418 |
| miR-501-3p | 3 | 4 | 1.32 | 0.295418 |
| miR-7a-1-3p | 3 | 4 | 1.32 | 0.295418 |
| miR-124-3p | 0 | 1 | 3.76 | 0.292546 |
| miR-1291 | 0 | 1 | 3.76 | 0.292546 |
| miR-135a-5p | 0 | 1 | 3.76 | 0.292546 |
| miR-139-3p | 0 | 1 | 3.76 | 0.292546 |
| miR-143-5p | 0 | 1 | 3.76 | 0.292546 |
| miR-183-3p | 0 | 1 | 3.76 | 0.292546 |
| miR-192-3p | 0 | 1 | 3.76 | 0.292546 |
| miR-210-3p | 0 | 1 | 3.76 | 0.292546 |
| miR-211-5p | 0 | 1 | 3.76 | 0.292546 |
| miR-328-5p | 0 | 1 | 3.76 | 0.292546 |
| miR-341-3p | 0 | 1 | 3.76 | 0.292546 |
| miR-378c | 0 | 1 | 3.76 | 0.292546 |
| miR-434-3p | 0 | 1 | 3.76 | 0.292546 |
| miR-505-5p | 0 | 1 | 3.76 | 0.292546 |
| miR-676-3p | 0 | 1 | 3.76 | 0.292546 |
| miR-6964-3p | 0 | 1 | 3.76 | 0.292546 |
| miR-743b-3p | 0 | 1 | 3.76 | 0.292546 |
| miR-872-3p | 0 | 1 | 3.76 | 0.292546 |
| miR-935 | 0 | 1 | 3.76 | 0.292546 |
| novel-miR-107-3p | 0 | 1 | 3.76 | 0.292546 |
| novel-miR-118-5p | 0 | 1 | 3.76 | 0.292546 |
| novel-miR-125-5p | 0 | 1 | 3.76 | 0.292546 |
| novel-miR-142-3p | 0 | 1 | 3.76 | 0.292546 |
| novel-miR-178-5p | 0 | 1 | 3.76 | 0.292546 |
| novel-miR-217-5p | 0 | 1 | 3.76 | 0.292546 |
| novel-miR-249-5p | 0 | 1 | 3.76 | 0.292546 |
| novel-miR-279-3p | 0 | 1 | 3.76 | 0.292546 |
| novel-miR-41-5p | 0 | 1 | 3.76 | 0.292546 |
| novel-miR-61-3p | 0 | 1 | 3.76 | 0.292546 |
| miR-32-5p | 9 | 9 | 0.99 | 0.292274 |
| miR-101a-3p | 3 | 0 | 0.09 | 0.290872 |
| miR-134-5p | 3 | 0 | 0.09 | 0.290872 |
| miR-19a-3p | 3 | 0 | 0.09 | 0.290872 |
| miR-5118 | 3 | 0 | 0.09 | 0.290872 |
| miR-370-3p | 8 | 2 | 0.25 | 0.288 |
| miR-465c-5p | 4 | 5 | 1.24 | 0.276828 |
| miR-744-5p | 4 | 5 | 1.24 | 0.276828 |
| miR-152-3p | 6 | 1 | 0.17 | 0.251896 |
| miR-1839-5p | 11 | 3 | 0.27 | 0.229864 |
| miR-431-5p | 45 | 20 | 0.44 | 0.227634 |
| miR-471-3p | 7 | 8 | 1.14 | 0.223094 |
| miR-493-5p | 75 | 36 | 0.48 | 0.21632 |
| miR-18a-5p | 26 | 23 | 0.88 | 0.204682 |
| miR-365-3p | 18 | 6 | 0.33 | 0.203046 |
| miR-378a-5p | 14 | 14 | 0.99 | 0.1952556 |
| miR-136-5p | 9 | 10 | 1.10 | 0.1924834 |
| miR-154-3p | 14 | 4 | 0.28 | 0.1851752 |
| miR-154-5p | 35 | 30 | 0.85 | 0.1849504 |
| miR-490-3p | 4 | 0 | 0.07 | 0.1796258 |
| miR-100-5p | 2766 | 1642 | 0.59 | 0.1748468 |
| miR-542-5p | 54 | 44 | 0.81 | 0.1717422 |
| miR-137-3p | 7 | 1 | 0.14 | 0.1717356 |
| miR-410-3p | 47 | 20 | 0.42 | 0.166062 |
| miR-184-3p | 2 | 4 | 1.99 | 0.1605184 |
| miR-1a-3p | 2 | 4 | 1.99 | 0.1605184 |
| miR-202-5p | 2 | 4 | 1.99 | 0.1605184 |
| novel-miR-116-3p | 5 | 7 | 1.39 | 0.1529486 |
| miR-25-5p | 1 | 3 | 2.98 | 0.1484938 |
| miR-30c-2-3p | 1 | 3 | 2.98 | 0.1484938 |
| miR-463-5p | 1 | 3 | 2.98 | 0.1484938 |
| miR-590-5p | 1 | 3 | 2.98 | 0.1484938 |
| novel-miR-112-3p | 1 | 3 | 2.98 | 0.1484938 |
| miR-130a-3p | 15 | 4 | 0.26 | 0.1392196 |
| miR-323-3p | 15 | 4 | 0.26 | 0.1392196 |
| miR-374b-5p | 26 | 9 | 0.34 | 0.1375954 |
| miR-142a-3p | 8 | 10 | 1.24 | 0.1306806 |
| miR-496a-3p | 13 | 3 | 0.23 | 0.1244488 |
| miR-296-5p | 9 | 11 | 1.21 | 0.123091 |
| miR-10b-5p | 10 | 12 | 1.19 | 0.115725 |
| miR-17-5p | 10 | 12 | 1.19 | 0.115725 |
| miR-128-1-5p | 0 | 2 | 7.52 | 0.1118858 |
| miR-129-2-3p | 0 | 2 | 7.52 | 0.1118858 |
| miR-212-3p | 0 | 2 | 7.52 | 0.1118858 |
| miR-219b-5p | 0 | 2 | 7.52 | 0.1118858 |
| miR-294-3p | 0 | 2 | 7.52 | 0.1118858 |
| miR-30c-1-3p | 0 | 2 | 7.52 | 0.1118858 |
| miR-551b-3p | 0 | 2 | 7.52 | 0.1118858 |
| novel-miR-351-5p | 0 | 2 | 7.52 | 0.1118858 |
| novel-miR-428-3p | 0 | 2 | 7.52 | 0.1118858 |
| miR-324-5p | 5 | 0 | 0.05 | 0.1109268 |
| miR-802-5p | 5 | 0 | 0.05 | 0.1109268 |
| miR-328-3p | 25 | 8 | 0.32 | 0.1054392 |
| miR-30a-3p | 11 | 2 | 0.18 | 0.1045828 |
| miR-708-5p | 11 | 2 | 0.18 | 0.1045828 |
| miR-532-5p | 73 | 60 | 0.82 | 0.1021362 |
| miR-871-5p | 13 | 15 | 1.15 | 0.0955464 |
| miR-9-3p | 6 | 9 | 1.49 | 0.0858898 |
| miR-1198-5p | 3 | 6 | 1.99 | 0.0856612 |
| miR-429-3p | 3 | 6 | 1.99 | 0.0856612 |
| miR-146a-5p | 49 | 19 | 0.39 | 0.0835028 |
| miR-421-3p | 2 | 5 | 2.48 | 0.0795788 |
| novel-miR-133-3p | 2 | 5 | 2.48 | 0.0795788 |
| miR-485-5p | 9 | 1 | 0.11 | 0.0778332 |
| miR-490-5p | 17 | 4 | 0.23 | 0.0766834 |
| miR-200c-3p | 238 | 176 | 0.73 | 0.0737562 |
| miR-196a-5p | 18 | 20 | 1.10 | 0.0688424 |
| miR-340-3p | 1 | 4 | 3.97 | 0.066899 |
| miR-342-3p | 15 | 18 | 1.19 | 0.0554094 |
| miR-487b-3p | 87 | 37 | 0.42 | 0.0548392 |
| miR-574-3p | 32 | 32 | 0.99 | 0.0536342 |
| miR-148a-3p | 39 | 13 | 0.33 | 0.0506334 |
| miR-125a-5p | 55 | 50 | 0.90 | 0.0485432 |
| miR-214-5p | 3 | 7 | 2.32 | 0.0438826 |
| miR-106b-5p | 0 | 3 | 11.28 | 0.0427914 |
| miR-326-3p | 0 | 3 | 11.28 | 0.0427914 |
| miR-499-5p | 0 | 3 | 11.28 | 0.0427914 |
| miR-29b-3p | 65 | 25 | 0.38 | 0.04134 |
| miR-205-5p | 57 | 21 | 0.37 | 0.03994 |
| miR-126a-5p | 52 | 49 | 0.94 | 0.0343762 |
| miR-222-3p | 242 | 118 | 0.48 | 0.0326732 |
| miR-92b-3p | 28 | 31 | 1.10 | 0.0249008 |
| miR-339-5p | 4 | 9 | 2.23 | 0.0246344 |
| miR-93-5p | 137 | 113 | 0.82 | 0.0240874 |
| miR-369-5p | 18 | 3 | 0.17 | 0.0232772 |
| miR-411-5p | 151 | 67 | 0.44 | 0.022157 |
| miR-203-3p | 58 | 20 | 0.34 | 0.0214858 |
| miR-15b-5p | 19 | 24 | 1.25 | 0.018995 |
| miR-467a-5p | 2 | 7 | 3.48 | 0.01800832 |
| miR-19b-3p | 45 | 46 | 1.02 | 0.01648988 |
| miR-128-3p | 19 | 25 | 1.31 | 0.01226854 |
| miR-27a-3p | 813 | 580 | 0.71 | 0.00936624 |
| miR-27a-5p | 7 | 14 | 1.99 | 0.0085894 |
| miR-409-3p | 54 | 16 | 0.29 | 0.00729742 |
| let-7e-5p | 126 | 50 | 0.39 | 0.00679874 |
| miR-125b-5p | 212 | 94 | 0.44 | 0.0064165 |
| miR-486a-5p | 0 | 5 | 18.80 | 0.00625922 |
| miR-25-3p | 39 | 44 | 1.12 | 0.00616378 |
| miR-141-3p | 51 | 54 | 1.05 | 0.005904 |
| miR-486a-3p | 3 | 10 | 3.31 | 0.00513814 |
| miR-484 | 36 | 42 | 1.16 | 0.00506546 |
| miR-18a-3p | 13 | 22 | 1.68 | 0.00323442 |
| miR-145a-5p | 115 | 106 | 0.92 | 0.00318388 |
| miR-9b-3p | 18 | 28 | 1.54 | 0.001898698 |
| miR-20a-5p | 43 | 51 | 1.18 | 0.001614976 |
| miR-16-5p | 509 | 392 | 0.76 | 0.00122246 |
| miR-320-3p | 88 | 89 | 1.00 | 0.0011106 |
| miR-24-3p | 1406 | 1001 | 0.71 | 0.000774462 |
| miR-199a-5p | 157 | 144 | 0.91 | 0.000686604 |
| miR-146b-5p | 16 | 28 | 1.74 | 0.000665122 |
| let-7d-5p | 93 | 28 | 0.30 | 0.000475048 |
| miR-470-5p | 8 | 20 | 2.48 | 0.000397024 |
| miR-150-5p | 112 | 112 | 0.99 | 0.00034288 |
| miR-194-5p | 95 | 28 | 0.29 | 0.000298096 |
| miR-183-5p | 27 | 41 | 1.51 | 0.000234944 |
| miR-30d-5p | 501 | 398 | 0.79 | 0.000220632 |
| miR-130b-5p | 19 | 34 | 1.78 | 0.000138962 |
| miR-122b-5p | 19 | 0 | 0.01 | 0.000130132 |
| miR-31-3p | 19 | 0 | 0.01 | 0.000130132 |
| miR-223-3p | 47 | 61 | 1.29 | 0.000123146 |
| miR-23a-3p | 1116 | 830 | 0.74 | 6.91E-05 |
| miR-29a-3p | 125 | 37 | 0.29 | 3.42E-05 |
| miR-96-5p | 22 | 40 | 1.81 | 2.94E-05 |
| miR-135b-5p | 77 | 92 | 1.19 | 1.98E-05 |
| miR-423-3p | 215 | 204 | 0.94 | 1.36E-05 |
| miR-449a-5p | 30 | 50 | 1.66 | 1.17E-05 |
| miR-465c-3p | 26 | 0 | 0.01 | 4.46E-06 |
| miR-34c-3p | 18 | 40 | 2.21 | 2.29E-06 |
| miR-379-5p | 94 | 20 | 0.21 | 2.01E-06 |
| miR-98-5p | 72 | 12 | 0.17 | 1.81E-06 |
| miR-143-3p | 1176 | 919 | 0.78 | 1.47E-07 |
| let-7b-5p | 243 | 78 | 0.32 | 1.29E-07 |
| miR-31-5p | 85 | 13 | 0.15 | 6.02E-08 |
| miR-30b-5p | 469 | 420 | 0.89 | 4.68E-08 |
| miR-26a-5p | 2598 | 1901 | 0.73 | 3.74E-08 |
| let-7c-5p | 149 | 35 | 0.23 | 2.16E-08 |
| miR-99b-5p | 484 | 447 | 0.92 | 1.34E-09 |
| miR-21a-5p | 888 | 378 | 0.42 | 4.54E-10 |
| let-7a-5p | 1078 | 468 | 0.43 | 5.97E-11 |
| miR-99a-5p | 377 | 119 | 0.31 | 1.71E-11 |
| let-7i-5p | 328 | 96 | 0.29 | 8.03E-12 |
| miR-34b-3p | 114 | 163 | 1.42 | 4.26E-12 |
| miR-181a-5p | 2177 | 1703 | 0.78 | 6.86E-13 |
| miR-34a-5p | 451 | 134 | 0.30 | 2.85E-15 |
| miR-221-3p | 344 | 87 | 0.25 | 8.17E-16 |
| miR-369-3p | 149 | 16 | 0.11 | 1.51E-16 |
| miR-465a-3p | 0 | 41 | 154.14 | 5.88E-18 |
| miR-122-3p | 278 | 53 | 0.19 | 1.03E-18 |
| miR-191-5p | 1047 | 978 | 0.93 | 3.85E-20 |
| miR-192-5p | 428 | 102 | 0.24 | 3.93E-21 |
| miR-127-3p | 475 | 120 | 0.25 | 2.55E-21 |
| let-7g-5p | 482 | 96 | 0.20 | 7.64E-30 |
| let-7f-5p | 676 | 129 | 0.19 | 2.57E-43 |
| miR-126a-3p | 2598 | 2460 | 0.94 | 6.36E-51 |
| miR-451a | 3039 | 2826 | 0.92 | 5.88E-54 |
| miR-22-3p | 7047 | 2828 | 0.40 | 2.51E-89 |
| miR-92a-3p | 729 | 1190 | 1.62 | 4.28E-98 |
| miR-122-5p | 2786 | 400 | 0.14 | 1.68E-229 |

Table S3 Differentially expressed abmiRNAs in response to *Echinococcus multilocularis* infection

| miRNA | Read count in the control | Read count in the infected group | Fold change | *P* value | Mean Ct values of serum qPCR^1^ | |
| --- | --- | --- | --- | --- | --- | --- |
|  |  |  |  |  | Negative | Positive |
| let-7a-5p | 1078 | 468 | 0.43 | 5.97E-11 | / | / |
| let-7b-5p | 243 | 78 | 0.32 | 1.29E-07 | / | / |
| let-7c-5p | 149 | 35 | 0.23 | 2.16E-08 | / | / |
| let-7d-5p | 93 | 28 | 0.30 | 0.000475 | / | / |
| let-7f-5p | 676 | 129 | 0.19 | 2.57E-43 | / | / |
| let-7g-5p | 482 | 96 | 0.20 | 7.64E-30 | / | / |
| let-7i-5p | 328 | 96 | 0.29 | 8.03E-12 | / | / |
| miR-122-3p | 278 | 53 | 0.19 | 1.03E-18 | 26.7 | 29.6 |
| miR-122-5p | 2786 | 400 | 0.14 | 1.68E-229 | 19.9 | 29.3 |
| miR-122b-5p | 19 | 0 | 0.01 | 0.0001301 | / | / |
| miR-127-3p^2^ | 475 | 120 | 0.25 | 2.55E-21 | / | / |
| miR-192-5p | 428 | 102 | 0.24 | 3.93E-21 | 23.7 | 28.3 |
| miR-194-5p | 95 | 28 | 0.29 | 0.0002981 | / | / |
| miR-21a-5p | 888 | 378 | 0.42 | 4.54E-10 | 16.3 | 24.3 |
| miR-221-3p | 344 | 87 | 0.25 | 8.17E-16 | 31.8 | 32.9 |
| miR-22-3p | 7047 | 2828 | 0.40 | 2.51E-89 | 24.9 | 29.6 |
| miR-29a-3p | 125 | 37 | 0.29 | 3.42E-05 | 29.8 | 33.6 |
| miR-31-3p | 19 | 0 | 0.01 | 0.0001301 | / | / |
| miR-31-5p | 85 | 13 | 0.15 | 6.02E-08 | 26.0 | 30.6 |
| miR-34a-5p | 451 | 134 | 0.30 | 2.85E-15 | 21.5 | 31.1 |
| miR-34c-3p | 18 | 40 | 2.21 | 2.29E-06 | 24.9 | 23.4 |
| miR-369-3p | 149 | 16 | 0.11 | 1.51E-16 | 34.9 | 34.0 |
| miR-379-5p | 94 | 20 | 0.21 | 2.01E-06 | / | / |
| miR-465a-3p | 0 | 41 | 154.14 | 5.88E-18 | 30.8 | 29.9 |
| miR-465c-3p | 26 | 0 | 0.01 | 4.46E-06 | / | / |
| miR-470-5p | 8 | 20 | 2.48 | 0.000397 | / | / |
| miR-98-5p | 72 | 12 | 0.17 | 1.81E-06 | 17.9 | 25.2 |
| miR-99a-5p | 377 | 119 | 0.31 | 1.71E-11 | 29.8 | 30.1 |

Note: ^1^The qPCR results are shown in the Fig. 2A; ^2^miR-127-3p is excluded for assessing the status of *E. multilocularis* infection due to the inconsistence between qPCR and sequencing data (Fig. 1C). AbmiRNAs, Argonaute 2-binding miRNAs.

Table S4 Analysis of the sensitivity of circulating miRNA-targeted qPCR using recombinant plasmids

| Expected concentration (copies/μL) | miR-122-5p | | | miR-21a-5p | | | miR-192-5p | | |
| --- | --- | --- | --- | --- | --- | --- | --- | --- | --- |
|  | Mean Ct | SD | % CV | Mean Ct | SD | % CV | Mean Ct | SD | % CV |
| 100,000 | 22.5 | 0.1 | 0.5 | 19.2 | 0.06 | 0.3 | 24.0 | 0.2 | 1.0 |
| 10,000 | 26.4 | 0.4 | 1.4 | 22.5 | 0.2 | 0.8 | 27.7 | 0.01 | 0.06 |
| 1,000 | 29.3 | 0.3 | 0.9 | 25.8 | 0.4 | 1.5 | 31.6 | 0.05 | 0.2 |
| 100 | 33.4 | 0.1 | 0.3 | 29.6 | 0.3 | 1.2 | 35.8 | 0.3 | 0.8 |
| 10 | 36.3 | 0.2 | 0.7 | 32.8 | 0.04 | 0.1 | Not detected | 0.05 | 0.1 |

Table S5 Youden’s index analysis of miR-192-5p-targeted qPCR

| Minimum threshold value | Sensitivity | 1 - specificity | Youden’s index |
| --- | --- | --- | --- |
| -0.9992 | 1 | 1 | 0 |
| 0.0009 | 1 | 0.96 | 0.04 |
| 0.0061 | 1 | 0.92 | 0.08 |
| 0.0122 | 1 | 0.88 | 0.12 |
| 0.0214 | 1 | 0.84 | 0.16 |
| 0.0298 | 1 | 0.8 | 0.2 |
| 0.0301 | 1 | 0.76 | 0.24 |
| 0.0367 | 1 | 0.72 | 0.28 |
| 0.0556 | 1 | 0.68 | 0.32 |
| 0.0768 | 1 | 0.64 | 0.36 |
| 0.0866 | 1 | 0.6 | 0.4 |
| 0.0995 | 1 | 0.56 | 0.44 |
| 0.1285 | 1 | 0.52 | 0.48 |
| 0.157 | 1 | 0.48 | 0.52 |
| 0.171 | 1 | 0.44 | 0.56 |
| 0.1766 | 1 | 0.4 | 0.6 |
| 0.1839 | 1 | 0.36 | 0.64 |
| 0.1917 | 1 | 0.32 | 0.68 |
| 0.1966 | 0.96 | 0.32 | 0.64 |
| 0.2007 | 0.96 | 0.28 | 0.68 |
| 0.2235 | 0.96 | 0.24 | 0.72 |
| 0.2455 | 0.96 | 0.2 | 0.76 |
| 0.2498 | 0.96 | 0.16 | 0.8 |
| 0.2682 | 0.96 | 0.12 | 0.84 |
| 0.2963 | 0.96 | 0.08 | **0.88** |
| 0.321 | 0.92 | 0.08 | 0.84 |
| 0.3477 | 0.92 | 0.04 | **0.88** |
| 0.3671 | 0.88 | 0.04 | 0.84 |
| 0.3835 | 0.84 | 0.04 | 0.8 |
| 0.4449 | 0.8 | 0.04 | 0.76 |
| 0.5141 | 0.76 | 0.04 | 0.72 |
| 0.5397 | 0.72 | 0.04 | 0.68 |
| 0.55 | 0.68 | 0.04 | 0.64 |
| 0.5547 | 0.64 | 0.04 | 0.6 |
| 0.5731 | 0.6 | 0.04 | 0.56 |
| 0.6135 | 0.56 | 0.04 | 0.52 |
| 0.7048 | 0.52 | 0.04 | 0.48 |
| 0.7824 | 0.48 | 0.04 | 0.44 |
| 0.809 | 0.48 | 0 | 0.48 |
| 0.8316 | 0.44 | 0 | 0.44 |
| 0.8952 | 0.4 | 0 | 0.4 |
| 0.9642 | 0.36 | 0 | 0.36 |
| 0.9874 | 0.32 | 0 | 0.32 |
| 1.0828 | 0.28 | 0 | 0.28 |
| 1.2259 | 0.24 | 0 | 0.24 |
| 1.4349 | 0.2 | 0 | 0.2 |
| 1.6459 | 0.16 | 0 | 0.16 |
| 1.947 | 0.12 | 0 | 0.12 |
| 2.5619 | 0.08 | 0 | 0.08 |
| 4.2168 | 0.04 | 0 | 0.04 |
| 6.4954 | 0 | 0 | 0 |

Note: The largest Youden’s index is in bold. For miR-192-5p-targeted qPCR, the maximum Youden’s index is present twice. In this case, we preferred to higher specificity and thus chose 0.35 as a cutoff value.

Table S6 Youden’s index analysis of miR-122-5p-targeted qPCR

| Minimum threshold value | Sensitivity | 1 - specificity | Youden’s index |
| --- | --- | --- | --- |
| -0.9991 | 1 | 1 | 0 |
| 0.001 | 1 | 0.96 | 0.04 |
| 0.0013 | 1 | 0.92 | 0.08 |
| 0.0019 | 1 | 0.88 | 0.12 |
| 0.0025 | 1 | 0.84 | 0.16 |
| 0.0033 | 1 | 0.8 | 0.2 |
| 0.0038 | 1 | 0.76 | 0.24 |
| 0.0051 | 1 | 0.72 | 0.28 |
| 0.0073 | 1 | 0.68 | 0.32 |
| 0.0084 | 1 | 0.64 | 0.36 |
| 0.0097 | 1 | 0.6 | 0.4 |
| 0.0116 | 1 | 0.56 | 0.44 |
| 0.0135 | 1 | 0.52 | 0.48 |
| 0.0151 | 1 | 0.48 | 0.52 |
| 0.016 | 1 | 0.44 | 0.56 |
| 0.0169 | 1 | 0.4 | 0.6 |
| 0.0199 | 1 | 0.36 | 0.64 |
| 0.0232 | 1 | 0.32 | 0.68 |
| 0.025 | 1 | 0.28 | 0.72 |
| 0.0262 | 1 | 0.24 | 0.76 |
| 0.0268 | 1 | 0.2 | 0.8 |
| 0.0298 | 0.96 | 0.2 | 0.76 |
| 0.0346 | 0.96 | 0.16 | 0.8 |
| 0.0421 | 0.96 | 0.12 | **0.84** |
| 0.0518 | 0.92 | 0.12 | 0.8 |
| 0.0585 | 0.88 | 0.12 | 0.76 |
| 0.1038 | 0.84 | 0.12 | 0.72 |
| 0.1731 | 0.8 | 0.12 | 0.68 |
| 0.2012 | 0.76 | 0.12 | 0.64 |
| 0.2049 | 0.76 | 0.08 | 0.68 |
| 0.21 | 0.72 | 0.08 | 0.64 |
| 0.2348 | 0.68 | 0.08 | 0.6 |
| 0.3166 | 0.64 | 0.08 | 0.56 |
| 0.4231 | 0.6 | 0.08 | 0.52 |
| 0.7347 | 0.56 | 0.08 | 0.48 |
| 1.2183 | 0.52 | 0.08 | 0.44 |
| 1.4847 | 0.52 | 0.04 | 0.48 |
| 1.5727 | 0.48 | 0.04 | 0.44 |
| 1.6826 | 0.44 | 0.04 | 0.4 |
| 1.8341 | 0.4 | 0.04 | 0.36 |
| 1.9548 | 0.36 | 0.04 | 0.32 |
| 2.1066 | 0.32 | 0.04 | 0.28 |
| 2.7218 | 0.28 | 0.04 | 0.24 |
| 3.7415 | 0.24 | 0.04 | 0.2 |
| 4.2622 | 0.24 | 0 | 0.24 |
| 6.0944 | 0.2 | 0 | 0.2 |
| 9.8426 | 0.16 | 0 | 0.16 |
| 12.9666 | 0.12 | 0 | 0.12 |
| 14.3108 | 0.08 | 0 | 0.08 |
| 18.0474 | 0.04 | 0 | 0.04 |
| 22.6442 | 0 | 0 | 0 |

Note: The largest Youden’s index is in bold.

Table S7 Youden’s index analysis of miR-21a-5p-targeted qPCR

| Minimum threshold value | Sensitivity | 1 - specificity | Youden’s index |
| --- | --- | --- | --- |
| -0.9997 | 1 | 1 | 0 |
| 0.0005 | 1 | 0.96 | 0.04 |
| 0.0008 | 1 | 0.92 | 0.08 |
| 0.0012 | 1 | 0.88 | 0.12 |
| 0.0019 | 1 | 0.84 | 0.16 |
| 0.0023 | 1 | 0.8 | 0.2 |
| 0.0024 | 1 | 0.76 | 0.24 |
| 0.0027 | 1 | 0.72 | 0.28 |
| 0.0032 | 1 | 0.68 | 0.32 |
| 0.0037 | 1 | 0.64 | 0.36 |
| 0.0039 | 1 | 0.6 | 0.4 |
| 0.0046 | 1 | 0.56 | 0.44 |
| 0.0054 | 1 | 0.52 | 0.48 |
| 0.0078 | 1 | 0.48 | 0.52 |
| 0.0138 | 1 | 0.44 | 0.56 |
| 0.0183 | 1 | 0.4 | 0.6 |
| 0.0216 | 1 | 0.36 | 0.64 |
| 0.0251 | 1 | 0.32 | 0.68 |
| 0.0269 | 1 | 0.28 | 0.72 |
| 0.0282 | 1 | 0.24 | 0.76 |
| 0.0288 | 1 | 0.2 | 0.8 |
| 0.03 | 0.96 | 0.2 | 0.76 |
| 0.0358 | 0.92 | 0.2 | 0.72 |
| 0.0501 | 0.92 | 0.16 | 0.76 |
| 0.0593 | 0.92 | 0.12 | 0.8 |
| 0.0818 | 0.92 | 0.08 | 0.84 |
| 0.13 | 0.92 | 0.04 | **0.88** |
| 0.1613 | 0.88 | 0.04 | 0.84 |
| 0.1892 | 0.84 | 0.04 | 0.8 |
| 0.2183 | 0.8 | 0.04 | 0.76 |
| 0.2548 | 0.76 | 0.04 | 0.72 |
| 0.317 | 0.76 | 0 | 0.76 |
| 0.3916 | 0.72 | 0 | 0.72 |
| 0.4399 | 0.68 | 0 | 0.68 |
| 0.6527 | 0.64 | 0 | 0.64 |
| 0.9298 | 0.6 | 0 | 0.6 |
| 1.0904 | 0.56 | 0 | 0.56 |
| 1.2025 | 0.52 | 0 | 0.52 |
| 1.3215 | 0.48 | 0 | 0.48 |
| 1.5003 | 0.44 | 0 | 0.44 |
| 1.7686 | 0.4 | 0 | 0.4 |
| 2.0079 | 0.36 | 0 | 0.36 |
| 2.2284 | 0.32 | 0 | 0.32 |
| 2.7627 | 0.28 | 0 | 0.28 |
| 3.6125 | 0.24 | 0 | 0.24 |
| 4.2679 | 0.2 | 0 | 0.2 |
| 4.6386 | 0.16 | 0 | 0.16 |
| 5.3356 | 0.12 | 0 | 0.12 |
| 7.2647 | 0.08 | 0 | 0.08 |
| 13.133 | 0.04 | 0 | 0.04 |
| 18.5703 | 0 | 0 | 0 |

Note: The largest Youden’s index is in bold.

Table S8 Comparison of the established qPCR approaches targeting individual circulating miRNAs molecules and their combinations in discriminating *E. multilocularis*-infected mice from healthy mice

|  | miR-192-5p | miR-122-5p | miR-21a-5p | miR-192-5p + miR-122-5p | miR-192-5p + miR-21a-5p | miR-122-5p + miR-21a-5p | miR-192-5p + miR-  122-5p + miR-21a-5p |
| --- | --- | --- | --- | --- | --- | --- | --- |
| miR-192-5p | / | *P* = 0.8412 | *P* = 0.5428 | *P* > 0.9999 | *P* > 0.9999 | *P* > 0.9999 | *P* > 0.9999 |
| miR-122-5p | / | / | *P* = 0.8773 | *P* = 0.8397 | *P* = 0.8397 | *P* = 0.8397 | *P* = 0.8397 |
| miR-21a-5p | / | / | / | *P* = 0.6818 | *P* = 0.6818 | *P* = 0.6818 | *P* = 0.6818 |
| miR-192-5p + miR-122-5p | / | / | / | / | *P* > 0.9999 | *P* > 0.9999 | *P* > 0.9999 |
| miR-192-5p + miR-21a-5p | / | / | / | / | / | *P* > 0.9999 | *P* > 0.9999 |
| miR-122-5p + miR-21a-5p | / | / | / | / | / | / | *P* > 0.9999 |
| miR-192-5p + miR-122-5p  + miR-21a-5p | / | / | / | / | / | / | / |

Note: ‘/’, not applicable.
